# Synthesis of Natural and Sugar-Modified Nucleosides Using the Iodine/Triethylsilane System as *N*-Glycosidation Promoter

**DOI:** 10.3390/ijms25169030

**Published:** 2024-08-20

**Authors:** Martina Cimafonte, Anna Esposito, Maria De Fenza, Francesco Zaccaria, Daniele D’Alonzo, Annalisa Guaragna

**Affiliations:** 1 Department of Chemical Sciences, University of Naples Federico II, I-80126 Naples, Italy; martinacimafonte7@gmail.com (M.C.); maria.defenza@unina.it (M.D.F.); francesco.zaccaria@unina.it (F.Z.); annalisa.guaragna@unina.it (A.G.); 2 Department of Chemical, Materials and Production Engineering, University of Naples Federico II, I-80125 Naples, Italy; anna.esposito5@unina.it

**Keywords:** nucleosides, *N*-glycosidation, stereoselectivity, iodine/triethylsilane, glycosyl iodide, anomerization, apricitabine

## Abstract

The reagent system based on the combined use of Et_3_SiH/I_2_ acts as an efficient *N*-glycosidation promoter for the synthesis of natural and sugar-modified nucleosides. An analysis of reaction stereoselectivity in the absence of C2-positioned stereodirecting groups revealed high selectivity with six-membered substrates, depending on the nucleophilic character of the nucleobase or based on anomerization reactions. The synthetic utility of the Et_3_SiH/I_2_-mediated *N*-glycosidation reaction was highlighted by its use in the synthesis of the investigational drug apricitabine.

## 1. Introduction

**Scheme 1:**
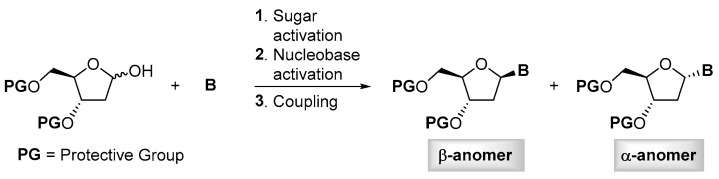
General scheme of the *N*-glycosidation reaction.

Whether in their monomeric or oligomeric form, nucleosides represent key biomolecules involved in a wide variety of research areas, spanning from chemistry to biology and from biotechnology to medicine [1,2,3,4,5]. Nucleosides have been the cornerstone of antiviral and anticancer therapy over the past five decades [6,7]. From early and well-known examples (zidovudine, lamivudine, acyclovir) up to the latest drugs (sofosbuvir, remdesivir, molnupiravir), nucleosides have become the standards of care for the treatment of many long-standing deadly diseases [8], while acting as first-line therapies for emerging infections of concern [9,10]. On the other hand, oligonucleotides are currently recognized as being among the most promising and versatile tools in modern drug discovery and diagnostics, aimed at the treatment, prevention and detection of various pathological conditions [11,12]. As illustrative examples in the field, mRNA vaccines have emerged as highly effective alternatives to conventional vaccines [13], boosted by the exceptional performance of Comirnaty (Pfizer-BioNTech) and Spikevax (Moderna) in counteracting the spread of COVID-19 [14,15]. Many of these notable successes have been achieved through the strategic replacement of natural entities with biomimetic analogues, built to resemble the structure of their physiological counterparts while altering (silencing or amplifying) their functions [16,17]. The demand for diverse nucleoside structures with unique chemical properties has driven intense efforts aimed at the development of efficient synthetic procedures, enabling their preparation in large amounts with high purity levels [18,19,20,21,22,23,24]. A crucial aspect of nucleoside synthesis focuses on the development of new protocols for the *N*-glycosylation reaction (Scheme 1). The latter is based on the formation of an *N*-glycosidic bond, achieved by a coupling reaction between a sugar and a nucleobase, both suitably activated [25].

**Scheme 2:**
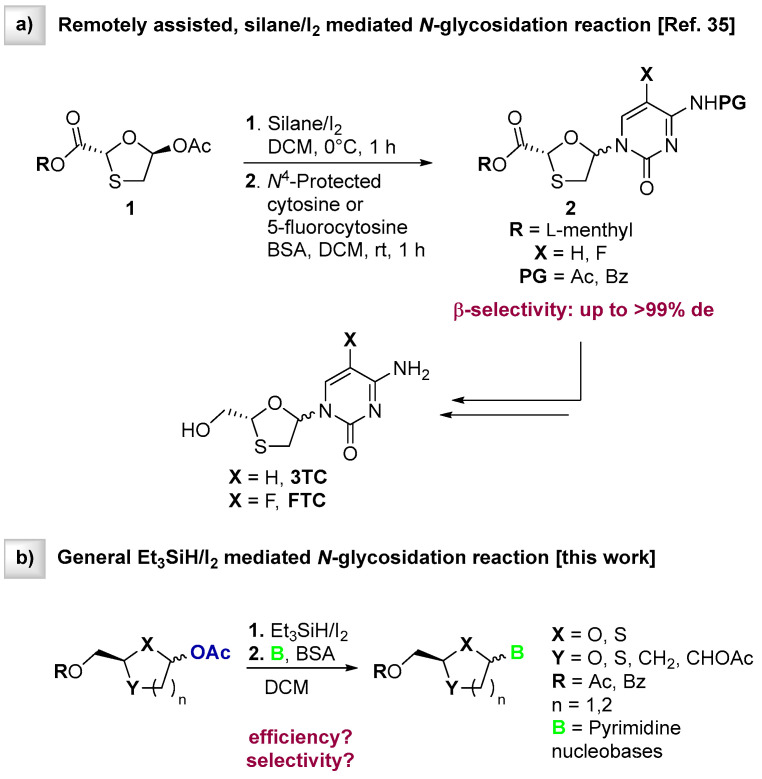
Silane/I_2_-mediated *N*-glycosidation reactions.

Considering that bioactive nucleosides are typically those with a β-configuration at their anomeric centre (i.e., the nucleobase and the hydroxymethyl group are involved in a *cis* stereochemical relationship), most *N*-glycosylation methods focus on stereoselective reactions toward β-nucleosides, regardless of their belonging to the d- or l-series. However, while synthetic strategies, from the earliest Vorbrüggen reaction [25] up to the recent Au-catalyzed [26] or epoxide-based methods [27], typically achieve β-selectivity by exploiting neighbouring group participation at the C2 position [28], the search for stereoselective approaches is much more challenging in the case of the synthesis of 2′-deoxynucleosides and their analogues. Only a few methods able to fulfil this purpose have been devised so far, based on remote stereocontrol [29], in situ complexation [30,31], S_N_2 reactions [32] and anomerization processes [33,34]. In this field, we have provided an example of a remotely controlled *N*-glycosidation reaction [35] using the well-known Et_3_SiH/I_2_ and PHMS/I_2_ systems [36,37], as exemplified by the coupling reaction of oxathiolane **1** with (5-fluoro)-cytosine *en route* to the synthesis of the antiretroviral drugs lamivudine (3TC) and emtricitabine (FTC) (Scheme 2a). The *N*-glycosidation reaction provided excellent results in terms of chemical efficiency and β-l-stereoselectivity (up to >99% de) as a result of the remote assistance of the menthyl ester group [38] (Scheme 2a). In addition, the nature of the protective group at the N4 position of cytosine or 5-fluorocytosine was demonstrated to have a deep influence on the stereoselective outcome of the reaction, as the electron-withdrawing group dramatically improved β- l-selectivity.

Based on these preliminary data, our ongoing efforts aimed to achieve selective transformations from halogen-containing substrates [39,40,41,42,43,44] led us to explore the stereoselective potential of the Et_3_SiH/I_2_ system in nucleoside synthesis beyond participating effects of the substrate. Particularly, the Et_3_SiH/I_2_-mediated *N*-glycosidation reaction has been studied using a variety of sugars and sugar-like substrates, lacking hydroxyl groups at the C2 and/or C3 positions (Scheme 2b). The last ones have been prepared by exploiting known synthetic procedures or slightly modified to improve their reaction yields (see Supplementary Materials for synthetic details). These compounds represent useful substrates for preparing modified nucleosides of biological/pharmacological interest [45], either to be employed as such or within more complex oligonucleotide structures [33,46]. Various pyrimidine nucleobases have been introduced to probe their influence on the stereoselective outcome of the reaction [35].

## 2. Results and Discussion

**Scheme 3:**
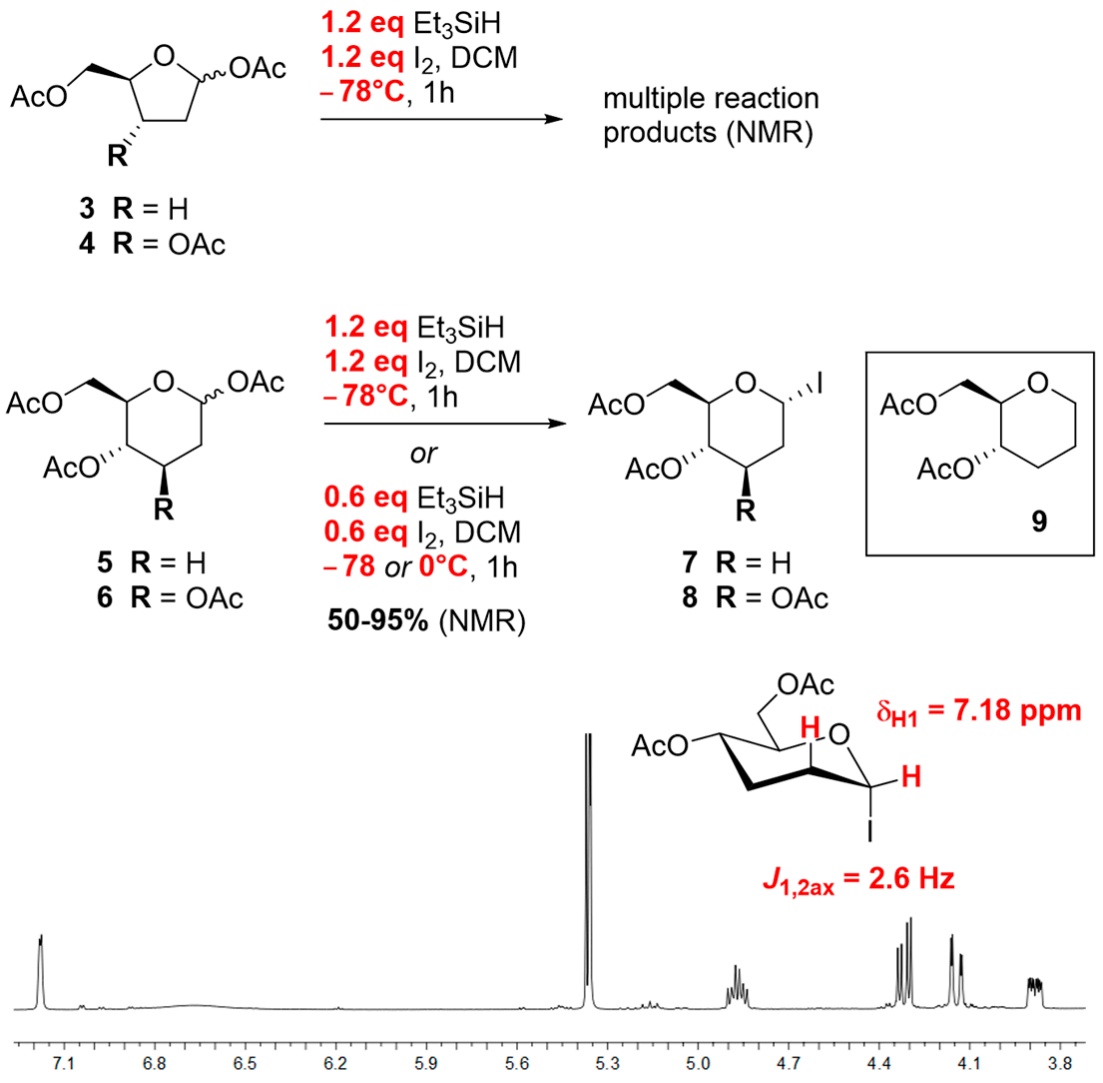
Et_3_SiI and HI as activating agents of glycoside acetates.

The general *N*-glycosidation procedure involved, as step 1 (Scheme 2b), the reaction of the deoxysugar substrate (an anomeric acetate) with the reactive species from the Et_3_SiH/I_2_ system (HI and Et_3_SiI), which was in turn obtained by premixing the two reagents and waiting for the resulting solution to discolour [47]. Attention was first focused on this activation step, to study its efficiency and stereoselectivity and to identify the actual iodinating agent. The treatment of five- and six-membered (di)deoxyglycosides **3–6** (Scheme 3) with the premixed Et_3_SiH/I_2_ rapidly (<1 h) led, as early as by −78 °C, to the disappearance of the starting sugars.

However, NMR analysis [48] of the crude mixture from furanosides **3** and **4** indicated the formation of a variety of species, and none of them were characterized by the presence of the typical downshielded anomeric protons (δ ~7 ppm) of glycosyl iodides [49]. On the other hand, the reaction of hexopyranosides **5** and **6** led to the exclusive formation of α-glycosyl iodides **7** and **8**, as demonstrated by their small *J*_1–2_ ^1^H NMR values (e.g., for compound **7**, *J*_1,2ax_ = 2.6 Hz, δ = 7.18 ppm; Scheme 3) [34]. To determine whether both in situ-generated HI and Et_3_SiI could work as activating agents, **5** was also treated with a substoichiometric amount (0.6 eq) of a 1:1 mixture of Et_3_SiH/I_2_. Under these conditions, at −78 °C, we only observed a ~50% conversion of **5** into the corresponding iodide **7**, while at 0 °C a much faster and complete conversion was found. This evidence demonstrated that both reagents enable glycoside activation, although Et_3_SiI, which is supposed to be the less reactive species [50], could be effectively exploited only at relatively high temperatures.

**Scheme 4:**
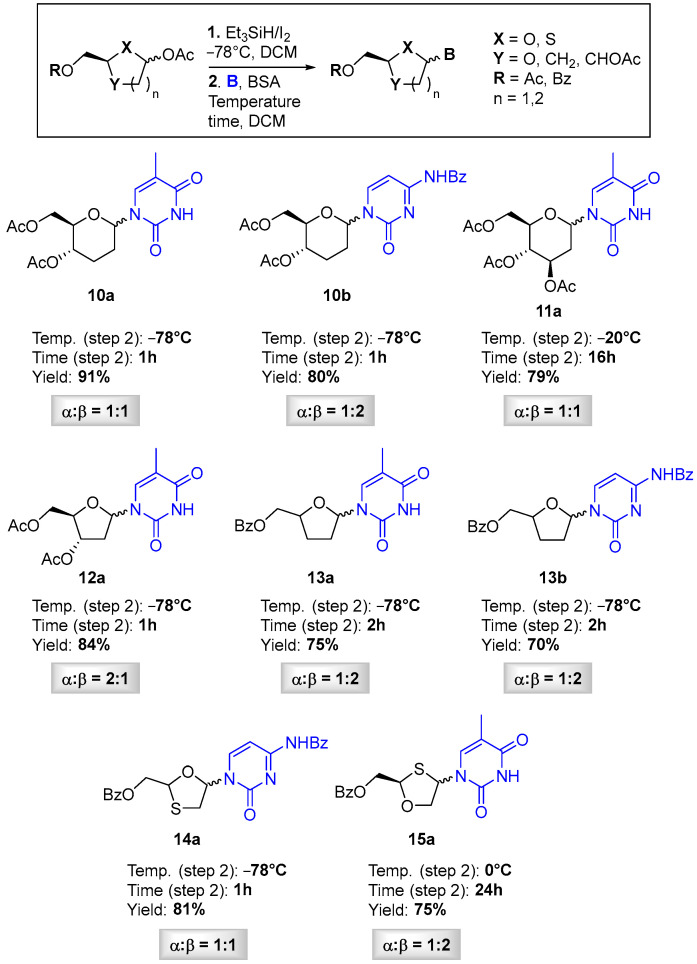
Et_3_SiH/I_2_-mediated *N*-glycosidation: preliminary screening. α:β ratios were assigned by comparative ^1^H NMR analysis with literature data (see Supplementary Materials for details).

**Scheme 5:**
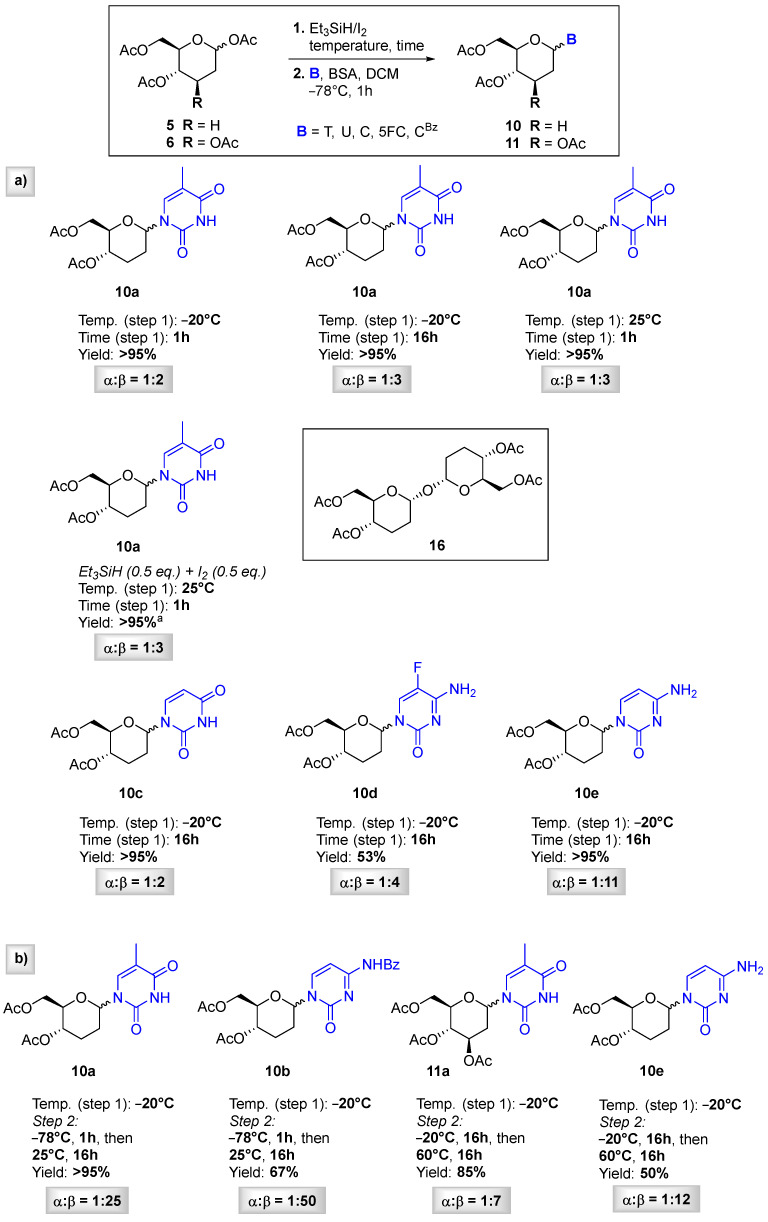
(**a**) Effect of temperature, time, nucleobase and eq. of activating reagents on the stereoselectivity of the *N*-glycosidation of hexopyranosides **5** and **6**. (**b**) Substrate-dependent effect of the temperature of step 2 on the stereoselective outcome of the *N*-glycosidations due to anomerization reactions. α:β ratios were assigned by comparative ^1^H NMR analyses with the literature data (see Supplementary Materials for details). ^a^ If a non-anhydrous reaction solvent was used, up to 90% of disaccharide **16** was recovered (see Supplementary Materials).

*N*-glycosidation reactions using thymine and (N4-benzoyl)cytosine as model nucleobases were then performed (Scheme 4 and Scheme 5; see Supplementary Materials for chemical structures and the synthesis of sugars and sugar-like substrates, Schemes S1–S5). After the iodination step was achieved (1.2 eq Et_3_SiH, 1.2 eq I_2_ at −78 °C) and TLC showed the disappearance of the starting glycoside acetate [51] (30 min), a solution of the nucleobase, previously silylated with *bis*-(trimethylsilyl)-acetamide (BSA) (DCM, 40 °C, 1–6 h), was added dropwise via a cannula under an argon flow. Glycosyl iodide consumption in favour of the corresponding nucleoside was commonly observed after 1–2 h. High-yielding conversions were observed in all cases (Scheme 4).

As an example, the reaction of dideoxyhexopyranoside **5** with thymine provided the corresponding nucleoside **10a** with a 91% yield. The same substrate, when reacted with persilylated N4-benzoylcytosine, provided deoxycytidine analogue **10b** with an 80% yield. The reaction of peracetylated 2-deoxyglucoside **6** with thymine required relatively higher temperatures (−20 °C), providing the corresponding nucleoside **11a** with a 79% yield. Thymidine **12a** and the nucleosides with a dideoxyribose (**13a** and **13b**) and an oxathiolane (**14a**) moiety were similarly obtained under the same conditions (70–84%). Conversely, oxathiolane nucleoside **15a** was obtained from the corresponding acetyl derivative only after keeping the reaction at 0 °C, and much more sluggishly [52] (24 h; Scheme 4). Looking at reaction stereoselectivity, only a very weak β-selectivity was observed under these conditions. As an example, thymine dideoxynucleoside **10a** was attained from the corresponding sugar substrate **5** with an α:β ratio of 1:1. The reaction of the same substrate with N4-benzoylcytosine furnished nucleoside **10b** with an α:β ratio of 1:2. Similar selectivities were observed for nucleosides bearing deoxyglucoside (**12a**), (di)deoxyribofuranoside (**13a** and **13b**) and oxathiolane moieties (**14a** and **15a**).

Looking for an improvement of the reaction stereoselectivity, *N*-glycosidation studies were performed by modifying parameters such as temperature, reaction time and the amount of activating reagents and/or nucleobases used (Scheme 5a). In most cases, hexopyranoside **5** was used as a model substrate. As an example, the treatment of **5** with Et_3_SiH/I_2_ was conducted, changing the step 1 temperature from −78 °C to −20 °C for 1 h or 16 h (Scheme 5a); afterwards, the reaction mixture was cooled to −78 °C and the addition of the persilylated nucleobase was performed at the same temperature. In this case, only a slight improvement in the α:β ratio (from 1:1 to 1:3) was observed in the reaction with thymine (compare with Scheme 4). A further increase in temperature of the iodination step up to rt did not further enhance the overall selectivity. Next, a substoichiometric amount (0.5 eq) of a 1:1 mixture of Et_3_SiH and I_2_ at room temperature was used. The α:β ratio still did not increase beyond 1:3. Reactions were eventually performed by changing the nucleobase (and keeping the temperature of step 1 at −20 °C). When uracil was used in place of thymine, the corresponding dideoxyuridine **10c** was obtained from **5** with an even lower selectivity (α:β = 1:2) than thymine nucleoside **10a**. The use of 5-fluorocytosine provided nucleoside **10d** with an increase in its α:β ratio to 1:4. Conversely, the same reaction carried out with cytosine provided the corresponding deoxycytidine analogue **10e** with an α:β ratio of 1:11. A further change in *N*-glycosidation conditions was eventually performed. As soon as the conversion of the starting acetates into the corresponding nucleosides was detected by TLC, we found that if the reaction mixtures were warmed to higher temperatures and left under stirring for longer times, the anomerization of α/β-nucleosides took place (Scheme 5b). As an example, the reaction of **5** with thymine, left at −78 °C for 1 h and then allowed to gradually reach room temperature for an additional 16 h, provided nucleoside **10a** with an α:β ratio of 1:25 (>95% yield). The α:β ratio obtained from the reaction of **5** with *N^4^*-benzoylcytosine, under the same conditions, was even higher (1:50, 67% after chromatographic purification). In the reaction of 2-deoxyglucose **6** with thymine, the anomeric α:β ratio increased from 1:1 to 1:7 in the formation of **11a**, but only after the reaction temperature was raised to 60 °C for 16 h. The same temperature did not significantly increase the α:β ratio in the reaction of **5** with cytosine (from 1:11 to 1:12; compare with Scheme 4).

**Scheme 6:**
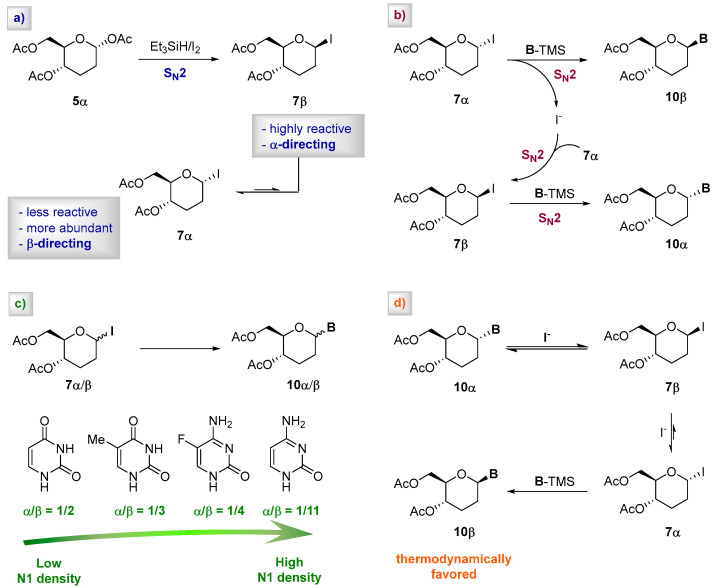
Stereoselectivity of the *N*-glycosidation reaction: mechanistic hypotheses.

The data reported in Scheme 5 deserve some comments. Starting from the observation that reactions with glycosyl iodides typically proceed under S_N_2 conditions [49], the presence of α/β anomeric mixtures of nucleosides demonstrate that the α-oriented glycosyl iodide **7α** is not the only reactive species in the solution. Indeed, even though an NMR analysis at room temperature indicated the presence of **7α** as the only anomeric glycosyl iodide, the occurrence at lower temperatures of the more reactive [53] β-glycosyl iodide **7β**, obtained under S_N_2 conditions from glycoside acetate **5α**, is expected (Scheme 6a).

This hypothesis could explain why, in the first trials, a complete lack of stereoselectivity was observed (Scheme 4). At higher temperatures, the formation of a certain amount of iodide **7β** by the anomerization of **7α** can take place as long as the reaction proceeds, considering that the concentration of iodide ion released from the substrate progressively increases (Scheme 6b).

While the overall stereoselectivity of the reaction was primarily influenced by the presence of **7β**, a strong nucleophile effect further affecting the α:β ratio was also observed. It was closely dependent on the electron density of the nucleobase (Scheme 6c). The highest β-selectivity was achieved with cytosine, which has the highest electron density at its N1 position. On the other hand, a linear decrease in selectivity was found as the electron density at N1 decreased, from 5-fluorocytosine to thymine and uracil [54] (Scheme 5 and Scheme 6).

Fruitful for a mechanistic analysis, we also observed that, when not strictly anhydrous conditions were used during the iodination step, the reaction smoothly proceeded to the undesired α,α-disaccharide **16** (Scheme 5a), which was detected as the only stereoisomer by NMR analysis (see Supplementary Materials). Accordingly, a reasonable hypothesis for the observed stereoselectivity assumes that weak nucleophiles (e.g., H_2_O) are able to discriminate between the more reactive α-directing β-glycosyl iodide and the more stable β-directing α-glycosyl iodide. With moderate nucleophiles (e.g., uracil, thymine and fluorocytosine), a low discrimination capacity is expected between the two glycosyl iodides, leading to low anomeric selectivity toward the β-isomers. With the stronger nucleophile cytosine, faster reactions and no discrimination at all between the two electrophilic species are conceivable. The attack will be therefore directed toward the most abundant species, i.e., α-glycosyl iodide **7α**, thus providing a high β-selectivity (Scheme 6c).

The results reported in Scheme 5 also demonstrate that hexopyranosides **5** and **6** are substrates for anomerization reactions. The increase in the β:α ratio demonstrates that no remote assistance of the C6-acetyl group occurs with these substrates. It is worth recalling that these substrates were already found to be amenable to anomerization reactions when using TfOTMS as an *N*-glycosidation promoter [34,55]. However, our conditions represent largely preferable alternatives to the above approach, as they provide higher β-selectivities while requiring lower reaction temperatures, thereby minimizing the formation of the side products encountered when using the previous method [34]. Compared to the TfOTMS-based reaction, the milder conditions required by the Et_3_SiH/I_2_ method clearly suggest an involvement of the iodide ion in promoting the anomerization rection, as depicted in Scheme 6d.

**Scheme 7:**
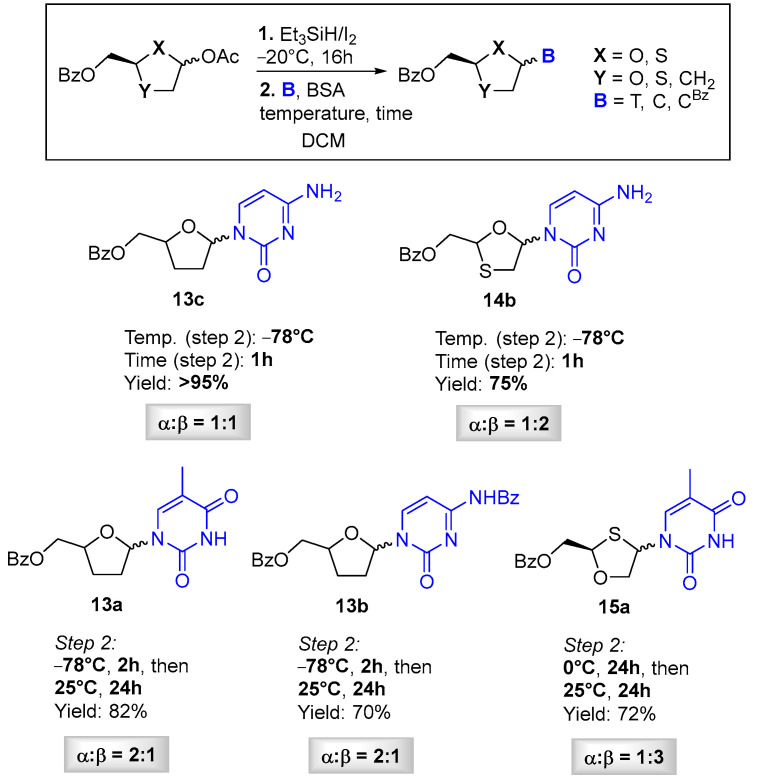
*N*-Glycosidation reactions of five-membered substrates.

The Et_3_SiH/I_2_-mediated *N*-glycosidation reaction was also tested on five-membered sugar-like substrates, modifying the reaction temperature, time, the amount of reagents used and the nucleobase (Scheme 7). The reaction again demonstrated high efficiency with all substrates (reaction yields: from 70 to >95%). Regarding reaction stereoselectivity, no relevant β-selectivity could be achieved with these substrates (α:β from 1:1 to 1:2). An enhancement of the β:α ratios by anomerization in the reactions of five-membered sugar substrates was observed, although it was much lower. As an example, after oxathiolanyl thymidine **15a** was obtained (0 °C, 24 h, α:β = 1:2, Scheme 4), the reaction mixture was left at room temperature for an additional 24 h, with an increase in the α:β ratio only up to 1:3 observed (Scheme 7). Under similar conditions, after dideoxy-thymidine or cytidine were formed (**13a** and **13b**) (−78 °C, 2 h), they were kept at rt for 24 h. Unexpectedly, an inversion in the α:β ratio (from 1:2 to 2:1) [56] was found in this case.

**Scheme 8:**
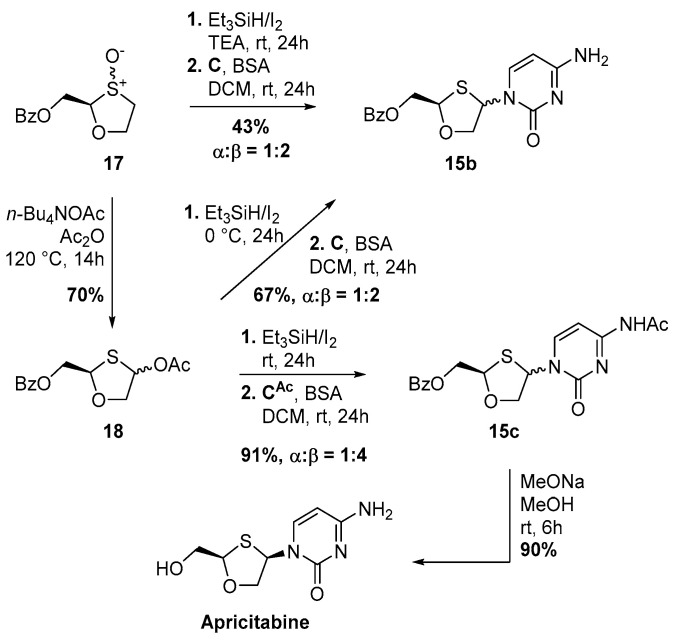
The use of the Et_3_SiH/I_2_-mediated *N*-glycosidation reaction in the synthesis of antiretroviral nucleoside apricitabine.

To highlight the usefulness of the Et_3_SiH/I_2_ reagent system in the synthesis of bioactive compounds, the method was eventually applied to improve the synthesis of the antiretroviral nucleoside apricitabine [57,58] (Scheme 8). We first studied if the enantiomerically enriched [59] sulfoxide **17** could serve as a substrate for Et_3_SiH/I_2_ *N*-glycosidation. However, after 1 h at −78 °C, the treatment of **17** with Et_3_SiH/I_2_ only provided the corresponding sulfide. As witnessed by the color change of the reaction mixture (from colorless to purple), the reaction most probably proceeds via formation of molecular iodine (see Supplementary Materials for mechanistic details, Scheme S6). On the other hand, the addition of an organic base (TEA) drove the reaction to the formation of the desired nucleoside **15b**, although in limited amounts (21%, 43% based on the recovery of the starting material) and with low β-selectivity. Conversely, the reaction of acetate **18** with cytosine ran much more efficiently (67%), although still with low selectivity (α:β = 1:2). When *N*^4^-acetylcytosine was employed, a higher stereoselectivity was observed, although it was still not excellent (α:β = 1:4). After the removal of the protective groups (MeONa), nucleoside **15c** could be converted into the desired apricitabine [58]. It is worth underlining that the above approach led to a β-selectivity higher than that reached by the synthetic method used for the industrial production of apricitabine (α:β = 1:2.9) [58].

## 3. Materials and Methods

### 3.1. General Methods

All moisture-sensitive reactions were performed under an argon atmosphere using oven-dried glassware. TLC (precoated silica gel plate F254, Merk Life Science S.r.l., Milan, Italy) was used to monitor reactions and compounds were detected by exposure to ultraviolet radiation and iodine vapour and by spraying a 5% ethanolic solution of sulfuric acid. Purifications of the compounds were performed by column chromatography (Merck Kieselgel 60, 70–230 mesh, Merk Life Science S.r.l., Milan, Italy). Combustion analyses were performed using a Flash Smart V elemental analyzer (Thermo Scientific Inc., Waltham, MA, USA). NMR spectrometers operating at 400 MHz (Bruker DRX, Bruker AVANCE, Bruker Corp., Billerica, MA, USA) or 500 MHz (Varian Inova equipped with a VnmrJ 4.0 software, Agilent Technologies, Santa Clara, CA, USA) were used to record NMR spectra. CDCl_3_ solutions were employed unless otherwise specified. Coupling constant values (J) were reported in Hz. ESI-MS spectra were recorded on a Shimadzu LCMS-8040 system with ESI interface, triple-quadrupole mass analyzer (Shimadzu Corporation, Kyoto, Japan) and Shimadzu LC-MS solution Workstation (version 5.97) software for data processing. Sugars and sugar-like substrates were prepared by exploiting known synthetic procedures [60,61,62,63,64,65,66,67,68,69,70,71,72,73,74] or slightly modified to improve their reaction yields (see Supplementary Materials for details; Schemes S1–S5). Copies of ^1^H and ^13^C NMR spectra for new compounds are provided in the Supplementary Material (Figures S1–S8).

### 3.2. N-Glycosidation Reactions

General procedure. Step 1: To a solution of I_2_ (0.12 mmol) in anhydrous DCM (0.5 mL), kept under an argon atmosphere, Et_3_SiH (0.12 mmol) was added at rt. The solution was stirred at the same temperature for 30 min. Then, the mixture was transferred via cannula into a solution of anomeric sugar acetate (0.10 mmol) in anhydrous DCM (0.5 mL) at the given temperature and stirred at the same temperature for 30 min. Step 2: *N*,*O*-*bis*(trimethylsilyl)acetamide (BSA) (0.32 mmol) was added to a suspension of the nucleobase (0.13 mmol) in anhydrous DCM (0.5 mL), kept under an argon atmosphere. The resulting suspension was warmed to 40 °C and stirred at the same temperature until a clear solution was formed (1–6 h). The mixture obtained in step 2 was then transferred via cannula into the solution obtained in step 1 at the given temperature and stirred therein for an appropriate amount of time under an argon atmosphere. Afterwards, the resulting solution was extracted with DCM and washed with brine and a few drops of an aqueous Na_2_S_2_O_3_ solution. The collected organic layers were dried (Na_2_SO_4_) and the solvent evaporated under reduced pressure. α:β ratios were assigned by a comparative ^1^H NMR analysis of the crude reaction mixtures with the literature data [75,76,77,78,79,80,81] (see Supplementary Materials for details).

## 4. Conclusions

The search for stereoselective methods capable of installing the *N*–glycosidic bond of natural and synthetic nucleosides without the involvement of stereodirecting groups still represents a challenging topic. Herein, a substantial extension of the *N*-glycosidation strategy relying on the combined use of Et_3_SiH/I_2_ has been performed. Various five- and six-membered deoxy and dideoxysugars and sugar-like substrates have been synthesized and tested in *N*-glycosidation reactions, with the aim of exploring the stereoselectivity of this reaction beyond the stereodirecting effects caused by neighbouring group participation. Our results indicate that Et_3_SiH/I_2_ is an excellent *N*-glycosidation reagent for the synthesis of natural and synthetic nucleosides, as it promotes, in most cases, stereoselective coupling reactions, especially in the case of six-membered sugar substrates. In this context, a “nucleophile effect” affecting the stereoselective outcome of the reactions has been found, and a mechanistic hypothesis has been formulated accordingly. The reaction has also been conveniently used for the synthesis of nucleoside structures with consolidated biological activity, such as apricitabine, with the finding that its β-selectivity was higher than that reported in the corresponding preparation processes used on an industrial scale. This result, combined with the low cost and high stability of the reagents, as well as with the high chemical efficiency of the reactions, contributes to making this methodology an attractive alternative to the existing *N*-glycosidation methods.

## Data Availability

The data presented in this study are available within the article and in the Supplementary Materials section.

## Supplementary Materials

The supporting information can be downloaded at: https://www.mdpi.com/article/10.3390/ijms25169030/s1.
