# Supplementary material for: Synthesis of Natural and Sugar-Modified Nucleosides Using the Iodine/Triethylsilane System as N-Glycosidation Promoter
Source: Int J Mol Sci. 2024 Aug 20;25(16):9030. doi: 10.3390/ijms25169030 (PMC11354600; doi:10.3390/ijms25169030)
Supplement: Supplementary file 1 [file ijms-25-09030-s001.zip › ijms-3135831-supplementary.pdf]

SUPPORTING INFORMATION

Synthesis of Natural and Sugar-Modified Nucleosides Using the Iodine/Triethylsilane System as *N*-Glycosidation Promoter

Martina Cimafonte [1,†], Anna Esposito [2,†], Maria De Fenza [1], Francesco Zaccaria [1], Daniele D'Alonzo [1,*] and Annalisa Guaragna [1]

[1] Department of Chemical Sciences, University of Naples Federico II, I-80126 Naples, Italy; martinacimafonte7@gmail.com (M.C.); maria.defenza@unina.it (M.D.F.); francesco.zaccaria@unina.it (F.Z.); annalisa.guaragna@unina.it (A.G.)

[2] Department of Chemical, Materials and Production Engineering, University of Naples Federico II, I-80125 Naples, Italy; anna.esposito5@unina.it

* Correspondence: dandalonzo@unina.it

† These authors contributed equally to this work.

TABLE OF CONTENTS

SYNTHESIS OF SUGARS AND SUGAR-LIKE SUBSTRATES	S2
N-GLYCOSIDATION REACTIONS	S7
SYNTHESIS OF APRICITABINE	S10
COPIES OF NMR SPECTRA	S12
BIBLIOGRAPHY	S20

General Methods

All moisture-sensitive reactions were performed under argon atmosphere using oven-dried glassware. TLC (precoated silica gel plate F254, Merck) were used to monitor reaction and compounds were detected by exposure to ultraviolet radiation, iodine vapor and spraying a 5% ethanolic solution of sulfuric acid. Purifications of compounds were performed by column chromatography (Merck Kieselgel 60, 70-230 mesh). Combustion analyses were performed using a Thermo Scientific Flash Smart V elemental analyser. NMR spectrometers operating at 400 MHz (Bruker DRX, Bruker AVANCE) or 500 MHz (Varian Inova equipped with a VnmrJ 4.0 software) were used to record NMR spectra. $CDCl_3$ solutions unless were employed otherwise specified. Coupling constant values (J) were reported in Hz. ESI-MS spectra were recorded on a Shimadzu LCMS-8040 system with ESI interface, triple-quadrupole mass analyzer and Shimadzu LC-MS solution Workstation (version 5.97) software for data processing.

Synthesis of sugars and sugar-like substrates

(4*S*,5*R*)-5-[(acetyloxy)methyl]oxolane-2,4-diyl diacetate (**4**). In line with the procedure of Marquez *et al.* [64], methylation of **19** with acetyl chloride (AcCl) and MeOH at 0°C for 16h followed by a subsequent acetylation of the crude residue (Ac_2O, Py) provided the α/β-mixture of pentofuranoside **21** (93% o.y.). Acetolysis of **21** (Ac_2O and H_2SO_4) at -20°C then yielded the desired 2-deoxypentofuranoside **4** as a mixture of anomers (99%; Scheme S1).

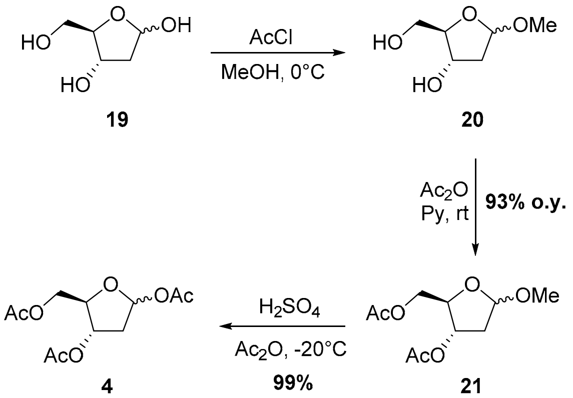

Scheme S1. Synthesis of 2-deoxyriboside **4** from 2-deoxyribose (**19**).

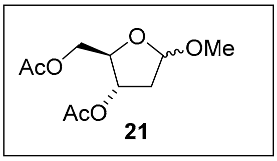

[(2*R*,3*S*)-3-(acetyloxy)-5-methoxyoxolan-2-yl]methyl (21). AcCl (0.32 mL, 4.50 mmol) was added in ice-cold MeOH (30 mL). The resulting mixture was warmed to rt and stirred at the same temperature for 30 min; then it was cooled to -20°C and after 30 min, 2-deoxy-d-ribose (**19**; 1.50 g, 11.2 mmol) was added. The resulting mixture was stirred at rt for 16h. After neutralization with solid sodium carbonate (0.30 g, 2.79 mmol), the solid was filtered off and the methanolic solution was evaporated under reduced pressure. The crude residue was then dissolved in EtOAc and filtered again and then the solvent was removed under reduced pressure. The resulting residue was treated with Ac_2O (5 mL) and pyridine (5 mL). Then the solution was stirred at rt for 16h. The solvents were evaporated under reduced pressure and chromatography of the crude residue over silica gel (hexane:EtOAc = 6:4) provided the pure **21** as a 1:1 α/β mixture (2.41 g, 93% o.y.). NMR data were fully in agreement with those reported elsewhere [65]. ^{1}H NMR (α/β = 1:1; 400 MHz): δ 1.89-1.98 (*m,* 0.5H), 2.01 (*s*, 3H), 2.02 (*s*, 3H), 2.06-2.15 (*m,* 0.5H), 2.28-2.36 (*m,* 1H), 3.27 (*s*, 1.5H), 3.33 (*s*, 1.5H), 4.02 (*dd*, J = 4.7, 11.4, 0.5H), 4.05-4.30 (*m*, 2.5H), 4.93-

4.99 (*m*, 0.5H), 5.03 (*d*, J = 3.9, 0.5H), 5.05-5.18 (*m*, 1H). ^{13}C NMR (α/β = 1:1; 100 MHz): ppm 20.6, 20.7, 20.8, 20.9, 38.7, 38.8, 55.0, 63.7, 64.6, 73.8, 74.8, 80.4, 81.6, 104.8, 105.4, 170.6, 170.8.

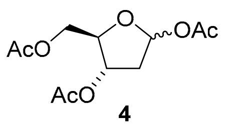

4

(4*S*,5*R*)-5-[(acetyloxy)methyl]oxolane-2,4-diyl diacetate (4). H_2SO_4 (50 µL) was added to a cold solution (-20°C) of **21** (2.1 g, 9.05 mmol) in Ac_2O (9 ml). The resulting mixture was stirred at the same temperature for 1h and then was extracted with AcOEt and washed with brine. The organic layers were dried (Na_2SO_4) and the solvent evaporated under reduced pressure. The resulting crude **4** (2.35 g, mixture of anomers; 99%) was used in the next step without further purification. NMR data were fully in agreement with those reported elsewhere [66]. α/β = 1:1. ^{1}H NMR (α/β = 1:1; 400 MHz): δ 2.05 (*s*, 1.5H), 2.06 (*s*, 1.5H), 2.07 (*s*, 1.5H), 2.08 (*s*, 1.5H), 2.09 (*s*, 1.5H), 2.10 (*s*, 1.5H), 2.12-2.20 (*m*, 0.5H), 2.25-2.37 (*m*, 0.5H), 2.43-2.55 (*m*, 1H), 4.07-4.20 (*m*, 1H), 4.20-4.32 (*m*, 1.5H), 4.41 (*q*, J = 3.6, 0.5H), 5.07-5.27 (*m*, 1H), 6.35 (*d*, J = 5.3 Hz, 1H), 6.39 (*dd*, J = 3.6, 5.9 Hz, 1H). ^{13}C NMR (α/β = 1:1; 100 MHz): ppm 20.6, 20.8, 21.1, 38.0, 38.2, 63.6, 64.0, 73.6, 73.8, 82.5, 83.0, 87.9, 98.2, 169.8, 170.1, 170.3, 170.4, 170.6.

(5*S*,6*R*)-6-[(acetyloxy)methyl]oxane-2,5-diyl diacetate (5). The sugar substrate **5** was synthesized starting from commercially available d-glucal **22** (Scheme S2). The latter was subjected to a Ferrier rearrangement (Koreeda variation [67]) involving catalytic I_2 and stoichiometric MeOH, to obtain methyl hexopyranoside **23** with moderate α-selectivity (α:β = 6:1) and in almost quantitative yield. Double bond reduction of **23** was then considered. The common hydrogenation procedure involving H_2 and Pd/C typically [68] provides variable amounts of the undesired pyran **9**. Conversely, the use of an excess of Raney-Ni in THF provided the expected hexopyranoside **24** in a good yield (80%) and with no traces of **9**. Finally, acetolysis of **24** (PTSA, AcOH and Ac_2O) gave, after 4h at 0°C, the desired hexopyranoside **5**, in a quantitative yield and with moderate α-selectivity (α:β = 5:1).

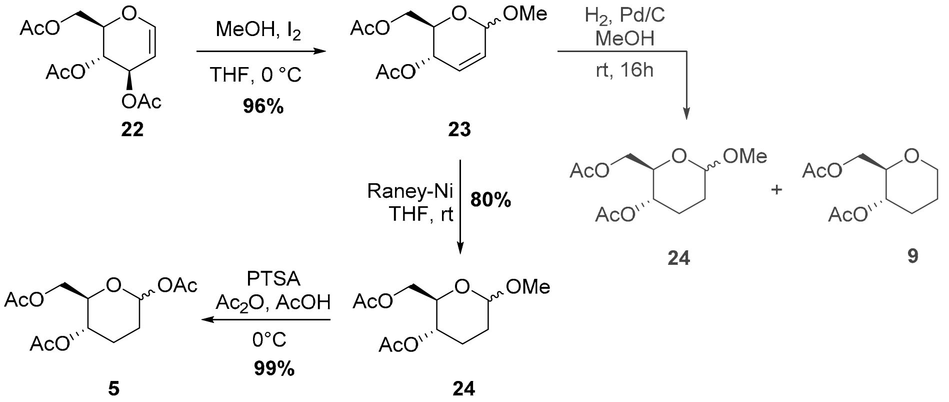

Scheme S2. Synthesis of dideoxy-hexopyranoside **5** from D-glucal **22**.

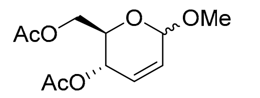

23

[(2*R*,3*S*)-3-(acetyloxy)-6-methoxy-3,6-dihydro-2*H*-pyran-2-yl]methyl acetate (23). To a cooled (0°C) solution of 3,4,6-tri-*O*-acetyl-d-glucal (**22**; 1.0 g, 3.68 mmol) in THF (20 mL), MeOH (0.15 mL, 3.68 mmol) and I_2 (188 mg, 0.74 mmol) were sequentially added. The resulting mixture was stirred at the same temperature for 16h and then was extracted with Et_2O and washed with brine. The organic layers were dried (Na_2SO_4) and the solvent evaporated under reduced pressure. The crude **23** (mixture of anomers, α/β = 6/1; 0.86 g, 96% yield) was used in the next step without further purification. NMR data were fully in agreement with those reported elsewhere [69]. ^{1}H NMR (major α-anomer; 400 MHz): δ 2.03 (s, 3H), 3.36 (*s*, 3H), 3.95-4.05 (*m*, 1H), 4.11 (*d*, J = 12.3, 1H), 4.19 (*dd*, J = 12.3, 5.4 Hz, 1H), 4.85 (*bs*, 1H), 5.26-

5.22 (*m*, 1H), 5.76 (*dd*, J = 10.6, 1.9, 1H), 5.85 (*bd*, J = 10.6 Hz, 1H). ^{13}C NMR (major α-anomer; 100 MHz): ppm 20.8, 20.9, 55.9, 62.9, 65.1, 66.7, 95.4, 127.6, 129.2, 170.3, 170.8.

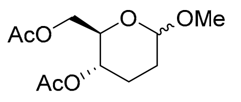

24

[(2*R*,3*S*)-3-(acetyloxy)-6-methoxyoxan-2-yl]methyl acetate (24). To a cooled (0 °C) suspension of Ni-Ra (1 g) in THF (4.4 mL), a solution of **23** (0.10 g, 0.41 mmol) in the same solvent (4.4 mL) was slowly added. The resulting suspension was warmed to rt for 16h. Then Ni-Ra was removed by filtration and the solvent was evaporated under reduced pressure. Chromatography of the crude residue over silica gel (hexane:acetone = 95:5) provided the pure **24** (81 mg; 80% yield). Data for **24**. NMR data were fully in agreement with those reported elsewhere [70]. ^{1}H NMR (major α-anomer; 400 MHz): δ 1.79-1.85 (m, 3H), 1.95-2.01 (m, 1H), 2.05 (s, 3H), 2.09 (s, 3H), 3.38 (s, 3H), 3.90 (*ddd*, J = 2.0, 4.9, 9.8, 1H), 4.11 (*dd*, *J* = 2.0, 11.7, 1H), 4.26 (*dd*, J = 4.9, 11.7, 1H), 4.70-4.76 (m, 2H). ^{13}C NMR (major α-anomer; 125 MHz): ppm 20.8. 21.1, 23.9, 28.6, 54.6, 63.2, 67.7, 68.5, 97.5, 170.0, 170.9. Data for pyran **9.** NMR data were fully in agreement with those reported elsewhere [71]. ^{1}H NMR (400 MHz): δ 1.42-1.52 (m, 1H), 1.68-1.79 (m, 2H), 2.04 (s, 3H), 2.10 (s, 3H), 2.24 (*dd*, J = 3.5, 12.4, 1H), 3.41 (*td*, J = 2.6, 11.8, 1H), 3.48 (*ddd*, J = 2.5, 5.2, 10.5, 1H), 3.99 (*dd*, *J* = 4.7, 11.4, 1H), 4.15 (*dd*, J = 2.5, 12.0, 1H), 4.20 (*dd*, J = 5.2, 12.0, 1H), 4.68 (*td*, J = 4.7, 10.5, 1H), ^{13}C NMR (125 MHz): ppm 20.9, 21.1, 24.9, 29,3, 63.8, 68.3, 68.4, 77.9, 170.1, 171.0.

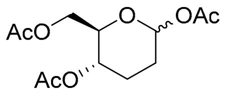

5

(5*S*,6*R*)-6-[(acetyloxy)methyl]oxane-2,5-diyl diacetate (5). To a cold (0°C) solution of **24** (0.22 g, 0.91 mmol) in Ac_2O/AcOH (2:1 v/v, 6.7 mL), *p*-toluenesulfonic acid (PTSA) was added in one portion (0.37 g, 1.95 mmol). The resulting mixture was stirred at the same temperature for 4h. Then it was extracted with EtOAc and washed with $NaHCO_3$. The organic layers were dried (Na_2SO_4) and the solvent was evaporated under reduced pressure to give **5** as a mixture of anomers (α/β = 5/1; 99%), which was used in the next step without further purification. NMR data were fully in agreement with those reported elsewhere [72]. ^{1}H NMR (major α-anomer; 500 MHz): δ 1.77-2.02 (*m*, 4H), 2.06 (*s*, 3H), 2.08 (*s*, 3H), 2.12 (*s*, 3H), 3.97- 4.03 (*m*, 1H), 4.11 (*dd*, J = 2.2, 12.2, 1H), 4.28 (*dd*, J = 4.8, 12.2, 1H), 4.77–4.84 (*td,* J = 5.2, 10.9, 1H), 6.14 (*bs*, 1H). ^{13}C NMR (major α-anomer; 100 MHz): δ 20.8, 21.0, 21.1, 23.6, 27.6, 62.7, 67.1, 70.8, 90.8, 169.3, 169.8, 170.8.

(4*R*,5*S*,6*R*)-6-[(acetyloxy)methyl]oxane-2,4,5-triyl triacetate (6). Standard per-*O*-acetylation of commercially available 2-deoxyglucose (**25**) directly gave hexopyranoside **6** as a mixture of anomers (99%; Scheme S3).

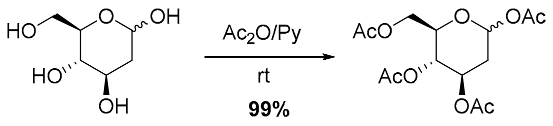

25 **6**

Scheme S3. Acetylation of 2-deoxyglucose.

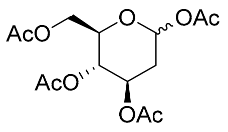

6

(4*R*,5*S*,6*R*)-6-[(acetyloxy)methyl]oxane-2,4,5-triyl triacetate (6). To a stirring solution of 2-deoxyglucose (**25**; 1.0 g, 3.44 mmol) in pyridine (10 mL), Ac_2O (5 mL) was added at room temperature. The resulting mixture was stirred for 16h and then the solvent was evaporated under reduced pressure to yield compound **6** as α/β=1:2 anomeric mixture (1.10 g, 99%). NMR data were fully in agreement with those reported elsewhere [73]. ^{1}H NMR (α/β = 1:2; 400 MHz): δ 1.65-1.79 (*m*,

0.66H), 1.80-1.84(*m*, 0.33H), 1.84-2.05 (*m*, 12H), 2.10-2.18 (*m*, 0.33H), 2.22 (*dd*, J = 5.0, 13.5, 0.66H), 3.63 (*ddd*, J = 2.0, 4.4, 9.2, 0.66H), 3.84-4.00 (m, 1.65H), 4.15-4.26 (m, 1H), 4.85-5.00 (*m*, 1.32H), 5.18-5.23 (*m*, 0.33H), 5.65 (*dd*, J = 2.3, 10.0, 0.66H), 6.18 (*d*, J = 2.5, 0.33H). ^{13}C NMR (α/β = 1:2; 100 MHz): ppm 20.5, 20.6, 20.7, 20.8, 33.7, 34.5, 61.6, 61.8, 67.8, 68.1, 68.5, 70.1, 72.7, 90.9, 168.6, 169.6, 169.9, 170.5.

[5-(Acetyloxy)oxolan-2-yl]methyl benzoate (29). Treatment of **26** with hydrogen peroxide (H_2O_2) and formic acid (HCOOH) at 60°C for 4h gave, in place of the expected epoxide, directly the lactone **27**. The latter was apparently the result of a cyclization of the *in situ* formed epoxide by the COOH group under acidic conditions. Subsequent benzoylation of the crude residue (BzCl/Py) provided ester **28** in a good 60% o.y. Chemoselective reduction (DIBAL-H) and subsequent acetylation of the crude residue then yielded the desired benzoate **29** as a mixture of anomers (86% o.y.; Scheme S4).

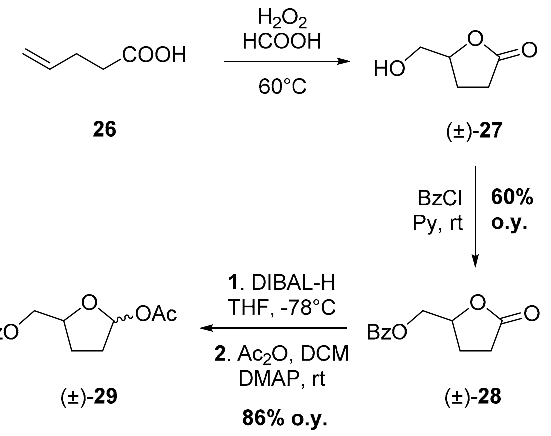

Scheme S4. Synthesis of benzoate **29** from 4-pentenoic acid.

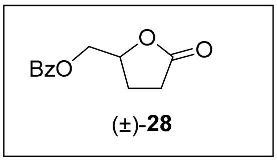

(5-Oxo-oxolan-2-yl)methyl benzoate (28). To a solution of 4-pentenoic acid (**26**; 1.0 g, 10.0 mmol) in H_2O (20 mL), H_2O_2 (7 mL, 30.5 mmol) and formic acid (22.6 mL) were added. The resulting solution was warmed to 60°C for 4h. Then the solvent was evaporated under reduced pressure. The crude residue was diluted with pyridine (15 mL), cooled at 0 °C and benzoyl chloride (1.5 mL, 13.0 mmol) was slowly added. The solution was stirred at the same temperature for 1h and then extracted with AcOEt and washed with brine. The organic layers were dried (Na_2SO_4) and the solvent evaporated under reduced pressure. Chromatography of the crude residue over silica gel (hexane:EtOAc = 6:4) provided the pure **28** (1.32 g, 60% o.y.) as a colourless oil. NMR data were fully in agreement with those reported elsewhere [74]. ^{1}H NMR (400 MHz): δ 2.08-2.15 (*m*, 1H), 2.40-2.45 (*m*, 1H), 2.56-2.65 (*m*, 2H), 4.44 (*dd*, J= 5.2, 12.2, 1H), 4.47 (*dd*, J = 3.3, 12.2, 1H), 4.82-4.90 (*m*, 1H), 7.45 (*t*, J = 7.4, 2H), 7.58 (*t*, J = 7.3, 1H), 8.03 (*d*, J = 7.3, 2H). ^{13}C NMR (100 MHz): ppm 23.8, 28.1, 65.6, 77.4, 128.4, 129.6, 129.8, 133.3, 166.0, 176.6.

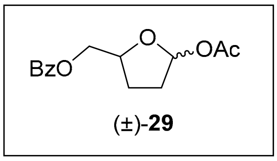

[5-(Acetyloxy)oxolan-2-yl]methyl benzoate (29). DIBAL-H (2.27 mL, 2.5 M solution in toluene) was added over one hour to a cooled (-78 °C) solution of **28** (0.53 g, 2.41 mmol) in anhydrous THF (10 mL). The solution was stirred at the same temperature for 30 min and then quenched with H_2O, extracted with AcOEt and washed with brine. The organic layers were dried (Na_2SO_4) and the solvent was evaporated under reduced pressure. To a solution of the resulting hemiacetal in CH_2Cl_2 (5 mL), Ac_2O (2 mL) and DMAP (19 mg, 0.16 mmol) were added. After stirring at rt for 1h, the solution was extracted with CH_2Cl_2 and washed with brine. The organic layers were dried (Na_2SO_4) and the solvent evaporated under

reduced pressure. Chromatography of the crude residue provided the pure **29** as mixture of diastereoisomers (0.53 g, 86% o.y., α/β = 1:1.5; colourless oil). ^{1}H NMR (α/β = 1:1.5; 400 MHz): 1.97 (*s*, 1.8H), 2.06 (*s*, 1.2H), 2.10-2.18 (*m*, 3H), 2.18-2.30 (*m*, 1H), 4.30-4.54 (*m*, 2.6H), 4.57-4.65 (*m*, 0.4H), 6.32 (*t*, J = 2.5, 0.6H), 6.39 (*d*, J = 4.1, 0.4 H), 7.42-7.50 (*m*, 2H), 7.53-7.60 (*m*, 1H), 8.03 (*d*, J = 7.1, 0.8H), 8.10 (*d*, J = 7.1, 1.2H). ^{13}C NMR (α/β = 1:1.5; 100 MHz): δ 21.0, 25.2, 31.7, 32.6, 66.2, 66.7, 77.7, 79.3, 98.9, 99.2, 100.0, 128.3, 128.4, 129.7, 129.8, 129.9, 133.1, 133.2, 166.5, 170.3. Elemental analysis calcd (%) for $C_{14}H_{16}O_5$: C 63.63, H 6.10; found: C 64.13, H 6.24.

[5-(Acetyloxy)-1,3-oxathiolan-2-yl]methyl benzoate (**32**). Coupling reaction of aldehyde **30** [59] with 1,4-dithiane-2,5-diol in pyridine at 60 °C for 1h provided oxathiolane **31** (63%). Eventually, acetylation under common conditions (Ac_2O/Py) yielded the desired [5-(acetyloxy)-1,3-oxathiolan-2-yl]methyl benzoate (**32**) in 45% yield.

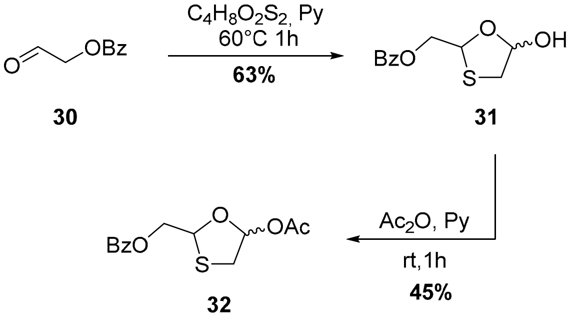

Scheme S5. Synthesis of oxathiolane **32** from ethylene glycol.

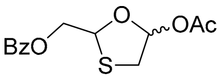

32

[5-(Acetyloxy)-1,3-oxathiolan-2-yl]methyl benzoate (32). To a solution of benzoyloxyacetaldehyde (**30**, 0.49 g, 3.0 mmol) in pyridine (50 ml), 1,4-dithiane-2,5-diol (0.23 g, 1.5 mmol) was added. The solution was warmed at 60-65 °C and was stirred at the same temperature for 1h. Then, the solvent was evaporated under reduced pressure. The crude residue (1.5 mmol) was diluted with pyridine (3.5 ml) and acetic anhydride (3.5 ml) was added at rt. The mixture was stirred at the same temperature for 1h. Then the solvent was evaporated under reduced pressure. Chromatography of the crude residue (hexane:AcOEt = 9:1) provided the pure **32** as a mixture of diastereoisomers (α:β = 2:1, 0.19 g, 45% yield). NMR data were fully in agreement with those reported elsewhere [75]. ^{1}H NMR (α:β = 2:1; 400 MHz): δ 2.09 (*s*, 0.99H), 2.11 (*s*, 1.98H), 3.12-3.28 (*m*, 0.66H), 3.35-3.42 (*m*, 1.32H) 4.42-4.68 (*m*, 2H), 5.64-5.72 (*m*, 1H), 6.64 (*d*, *J* = 4.6, 0.33H), 6.72 (*d*, J = 4.1, 0.66H), 7.45 (*t*, J = 7.9, 2H), 7.60 (*t*, J = 7.9, 1H), 8.05 (*d*, J = 7.9, 2H). ^{13}C NMR (α:β = 2:1; 100 MHz): ppm 21.1, 29.6, 37.5, 38.1, 66.1, 67.7, 83.3, 84.7, 99.2, 99.3, 127.9, 128.1, 129.4, 129.5, 129.8, 133.3, 166.0, 169.8.

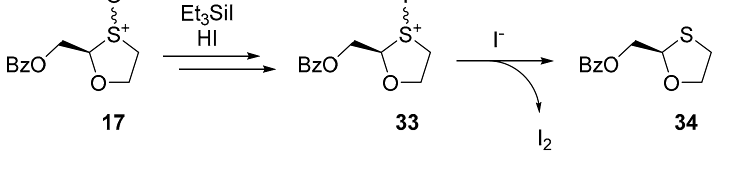

Scheme S6. Formation of sulfide **34** by treatment of **17** with Et_3SiH/I_2.

N-Glycosidation reactions

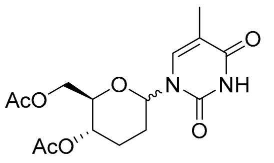

10a

{(2*R*,3*S*)-3-(acetyloxy)-6-[5-methyl-2,4-dioxo-3,4-dihydropyrimidin-1(2*H*)-yl]oxan-2-yl}methyl acetate (10a). DCM/MeOH = 98:2; 91-95% yield; see Schemes 4 and 5. NMR data were fully in agreement with those reported elsewhere [76]. ^{1}H NMR (α:β = 1:1; 400 MHz): δ 1.64 -1.85 (*m*, 2H), 1.95 (*s*, 3H), 1.98-2.04 (*m*, 1H), 2.07 (*s*, 1.5H), 2.08 (*s*, 1.5H), 2.09 (*s*, 1.5H), 2.10 (*s*, 1.5H), 2.30-2.40 (*m*, 1H), 3.78-3.89 (*m*, 0.5H), 4.16 (*dd*, J= 2.1, 12.2, 0.5H), 4.18-4.38 (*m*, 2H), 4.75 (*td,* J= 4.7, 10.2, 0.5H), 4.87 (*bs*, 0.5H), 5.75 (*dd*, J = 2.4, 10.7, 0.5H), 5.93 (*dd*, J = 3.5, 10.4, 0.5H), 7.18 (*s*, 1H). ^{13}C NMR (α:β = 1:1; 100 MHz): ppm 12.5, 12.6, 20.8, 21.0, 21.1, 21.2, 24.4, 24.9, 27.9, 29.3, 60.6, 62.9, 65.8, 66.5, 75.7, 76.6, 77.9, 81.3, 111.2, 111.5, 134.9, 135.2, 150.0, 150.3, 163.3, 163.9, 170.0, 170.2, 170.4, 170.8.

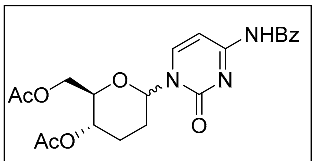

10b

{(2*R*,3*S*)-3-(acetyloxy)-6-[4-(benzoylamino)-2-oxopyrimidin-1(2*H*)-yl]oxan-2-yl}methyl acetate (10b). DCM/MeOH = 98:2; 67-80% yield; see Schemes 4 and 5. NMR data were fully in agreement with those reported elsewhere [76]. ^{1}H NMR (α:β = 1:2; 400 MHz): δ 1.71-1.88 (*m*, 2H), 2.11 (*s*, 1.98H), 2.12 (*s*, 1.98H), 2.15 (*s*, 0.99H), 2.17 (*s*, 0.99H), 2.31-2.43 (*m*, 2H), 3.87-3.95 (*m*, 0.66H), 4.22 (*dd*, J = 2.1, 12.2, 0.66H), 4.30-4.35 (*m*, 1.32H), 4.36-4.40 (*m*, 0.33H), 4.80 (*td*, J= 4.8, 10.4, 0.66H), 4.92 (*bs*, 0.33H), 5.88 (*d*, J= 10.3, 1H), 6.03 (*dd*, J= 1.6, 10.8, 0.33H), 7.55 (*t*, J= 7.8, 2H), 7.65 (*t*, J= 7.3, 1H), 7.92 (*d*, J= 7.3, 2H), 8.70 (*bs*, 1H). ^{13}C NMR (α:β = 1:2; 100 MHz): δ 20.7, 20.8, 21.0, 21.2, 27.8, 29.4, 30.2, 31.9, 61.5, 62.8, 65.8, 66.6, 75.7, 78.0, 79.2, 83.2, 127.6, 129.1, 132.8, 133.3, 142.6, 162.3, 170.0, 170.2, 170.5, 170.8.

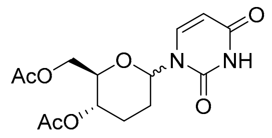

10c

{(2*R*,3*S*)-3-(acetyloxy)-6-[2,4-dioxo-3,4-dihydropyrimidin-1(2*H*)-yl]oxan-2-yl}methyl acetate (10c). DCM/MeOH = 98:2; >95% yield; see Scheme 5. NMR data were fully in agreement with those reported elsewhere [76]. ^{1}H NMR (α:β = 1:2; 400 MHz): δ 1.66-1.80 (*m*, 2H), 1.82-1.96 (*m*, 1H), 2.07 (*s*, 1.98H), 2.09 (*s*, 1.98H), 2.11 (*s*, 0.99H), 2.14 (*s*, 0.99H), 2.31-2.42 (*m*, 1H), 3.84 (*ddd*, J = 2.0, 5.3, 10.0, 0.66H), 4.12 (*dd*, J = 2.1, 12.4 Hz, 0.66H), 4.20-4.33 (*m*, 1.65H), 4.42 (*dd*, J = 6.4, 11.0 Hz, 0.33H), 4.66-4.76 (*m,* 0.66H), 4.87 (*bs*, 0.33H), 5.73 (*d*, J = 8.2, 0.66H), 5.78 (*d*, J = 7.9, 1H), 5.93 (*dd*, J = 3.4, 10.1, 0.33H), 7.39 (*d*, J = 8.2, 0.66H), 7.47 (*d*, J = 8.2, 0.33H). ^{13}C NMR (α:β = 1:2; 100 MHz): ppm 20.7, 20.8, 20.9, 21.2, 27.8, 30.3, 31.9, 34.9, 61.5, 62.7, 65.6, 66.4, 75.8, 77.9, 81.5, 102.7, 103.0, 139.3, 139.6, 149.9, 150.0, 163.1, 163.2, 170.0, 170.1, 170.5, 170.8.

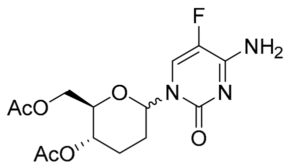

10d

{(2*R*,3*S*)-3-(acetyloxy)-6-[4-amino-5-fluoro-2-oxopyrimidin-1(2*H*)-yl]oxan-2-yl}methyl acetate (10d). DCM/MeOH = 98:2; >95% yield; see Scheme 5; colourless oil. ^{1}H NMR (α:β = 1:4; 400 MHz): δ 1.48-1.63 (*m*, 1H), 1.65-1.80 (*m*, 1H), 2.06 (*s*, 2.4H), 2.08 (*s*, 2.4H), 2.09 (*s*, 0.6H),), 2.11-2.24 (*m*, 1.6H), 2.26-2.38 (*m*, 0.8H), 3.75-3.93 (*m*, 0.8H), 4.15 (*d*, J = 12.2, 0.8H), 4.18-4.31 (*m*, 1.2H), 4.32-4.45 (*m*, 0.2H) 4.71 (*td*, J= 4.8, 10.6, 1H), 4.86 (*bs*, 0.2H), 5.76 (*d*, J = 10.4, 0.8H), 5.93 (*d*, J = 10.5, 0.2H), 7.48 (*d*, J = 6.0, 1H). 7.58 (*d*, J = 6.0, 1H). ^{13}C NMR (α:β = 1:4; 100 MHz): δ 20.8, 21.0, 21.2, 23.9, 25.3, 27.7, 29.8, 61.6, 62.8, 65.7, 66.6. 75.8, 77.9, 78.6, 82.6, 125.0, 125.3, 125.6, 135.3, 137.7, 151.9, 152.5, 156.4, 156.6, 156.9, 157.0, 170.0, 170.1, 170.45, 170.7. Elemental analysis calcd (%) for $C_{14}H_{18}FN_3O_6$: C 48.98, H 5.28, N 12.24; found: C 49.27, H 5.68, N 11.96.

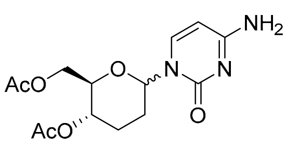

10e

{(2*R*,3*S*)-3-(acetyloxy)-6-[4-amino-2-oxopyrimidin-1(2*H*)-yl]oxan-2-yl}methyl acetate (10e). DCM/MeOH = 95:5; >95% yield; see Scheme 5; colourless oil. ^{1}H NMR (β-anomer; 400 MHz): δ 1.54-1.83 (*m*, 2H), 1.98-2.11 (*m*, 7H), 2.11-2.23 (*m*, 1H), 2.28-2.37 (*m*, 1H), 3.85 (*ddd*, J = 2.0, 5.0, 10.0, 1H),, 4.16 (*dd*, J = 1.9, 12.1, 1H), 4.25 (*dd*, J = 5.5, 12.1, 1H), 4.73 (*td*, J= 5.0, 10.3, 1H), 5.76 (*d*, J = 7.4, 1H), 6.17 (*dd*, J= 2.2, 10.4, 1H), 7.54 (*d*, J = 7.40, 1H). ^{13}C NMR (β-anomer; 100 MHz): ppm 20.9, 21.1, 27.9, 30.0, 62.9, 66.7, 78.1, 82.4, 95.8, 141.3, 153.4, 163.5, 170.1, 170.9. Elemental analysis calcd (%) for $C_{14}H_{19}N_{3}O_{6}$: C 51.69, H 5.89, N 12.92; found: C 51.90, H 5.87, N 12.84.

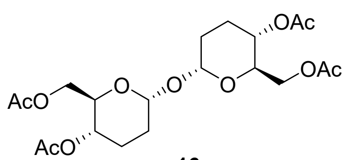

16

[(2*R*,3*S*,6*R*)-3-(acetyloxy)-6-({(2*R*,5*S*,6*R*)-5-(acetyloxy)-6-[(acetyloxy) methyl]oxan-2-yl}oxy)oxan-2-yl]methyl acetate (16). DCM/MeOH = 99:1; 90% yield; see Scheme 5; colourless oil. ^{1}H NMR (α,α- anomer; 500 MHz): δ 1.78-1.94 (*m*, 6H), 2.05 (*s*, 6H), 2.06-2.11 (*m*, 8H), 3.84-3.93 (*m*, 2H), 4.07 (*dd*, J = 2.2, 12.0, 2H), 4.24 (*dd*, J = 5.3, 12.0, 2H), 4.71-4.80 (*m*, 1H), 5.16 (*bs*, 2H). ^{13}C NMR (α,α- anomer; 100 MHz): ppm 20.7, 21.0, 23.7, 28.4, 63.1, 67.8, 69.1, 91.5, 169.9, 170.8. Elemental analysis calcd (%) for $C_{20}H_{30}O_{11}$: C 53.81, H 6.77; found: C 53.66, H 6.45. ESI-MS: calcd 447.18 ([M-H]$^{+}$); found 469.12 ([M-Na]$^{+}$), 485.16 ([M-K]$^{+}$).

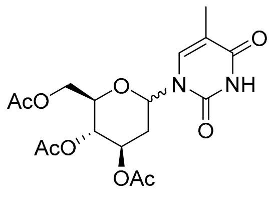

11a

(2*R*,3*S*,4*R*)-2-[(acetyloxy)methyl]-6-[5-methyl-2,4-dioxo-3,4-dihydropyrimidin-1(2*H*)-yl]oxane-3,4-diyl diacetate (11a). Hexane/EtOAc = 7:3; 79-85% yield; see Schemes 4 and 5. NMR data were fully in agreement with those reported elsewhere [77]. ^{1}H NMR (400 MHz): δ 1.95 (*s*, 3H), 2.03 (*s*, 3H), 2.07 (*s*, 3H), 2.35-2.48 (*m*, 2H), 4.25-4.37 (*m*, 2H), 5.04 (*t.* J= 9.6, 1H), 5.10-5.23 (*m*, 1H), 5.87 (*dd*, J = 2.2, 11.3, 1H), 7.18 (*s*, 1H). ^{13}C NMR (100 MHz): ppm 12.6, 35.2, 62.5, 68.2, 70.6, 75.0, 78.9, 111.9, 134.5, 149.7, 163.0, 169.87, 169.8, 170.6.

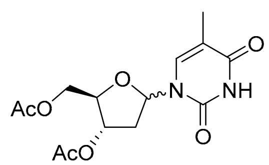

12a

{(2*R*,3*S*)-3-(acetyloxy)-5-[5-methyl-2,4-dioxo-3,4-dihydropyrimidin-1(2*H*)-yl]oxolan-2-yl}methyl acetate (**12a**). DCM/MeOH = 98:2; 84% yield; see Scheme 4. NMR data were fully in agreement with those reported elsewhere [78]. ^{1}H NMR (α:β = 2:1; 400 MHz): δ 1.94 (*s*, 0.99H), 1.95 (*s*, 1.8H), 2.13 (*s*, 3H), 2.15 (*s*, 3H), 2.19-2.29 (*m*, 1H), 2.46 (*ddd*, J= 1.9, 5.7, 14.2, 0.33H), 2.73-2.88 (*m*, 0.66H), 4.14-4.27 (*m*, 1.65H), 4.29-4.42 (*m*, 0.66H), 4.59 (*t.* J= 4.3, 0.66H), 5.16-5.27 (*m*, 1H), 6.26 (*dd*, J= 2.4, 7.3, 0.66H), 6.31 (*dd*, J= 5.9, 6.6, 0.33H), 7.27 (*s*, 0.33H), 7.30 (*s*, 0.66H), 8.00 (*bs*, 1H). ^{13}C NMR (α:β = 2:1; 125 MHz): ppm 12.7, 20.7, 20.8, 20.9, 37.5, 38.4, 63.7, 74.1, 74.3, 82.1, 84.3, 84.8, 86.7, 110.3, 111.5, 134.5, 135.2, 150.1, 163.3, 163.6, 169.8, 170.1, 170.3, 170.4.

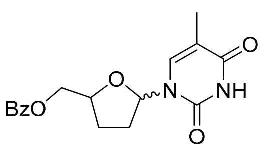

13a

{5-[5-methyl-2,4-dioxo-3,4-dihydropyrimidin-1(2*H*)-yl]oxolan-2-yl}methyl benzoate (**13a**). DCM/MeOH = 98:2; 75-82% yield; see Schemes 4 and 7. NMR data were fully in agreement with those reported elsewhere [79]. ^{1}H NMR (α:β = 1:2; 400 MHz): δ 1.71 (*s*, 0.99H), 1.94 (*s*, 0.99H), 1.93-2.13 (*m*, 0.66H), 1.93-2.13 (*m*, 0.66H), 2.16-2.32 (*m*, 0.33H), 2.38-2.61 (*m*, 0.33H), 4.25-4.48 (*m*, 0.99H), 4.53 (*dd,* J= 4.3, 0.66H), 4.66 (*dd*, J = 2.7, 11.9, 0.66H), 4.69-4.78 (*m*, 0.66H), 6.10 (*dd*, J = 4.6, 6.7, 0.66H), 6.14 (*t*, J = 5.4, 0.33H). 7.15 (*s*, 0.33H), 7.35 (*s*, 0.66H), 7.46 (*t*, J = 7.9, 2H), 7.51-7.63 (*m*, 1H), 7.97-8.12 (*m*, 2H). ^{13}C NMR (α:β = 1:2; 125 MHz): ppm 12.3, 12.6, 26.0, 26.6, 32.2, 32.3, 65.3, 66.2, 78.4, 78.8, 86.1, 87.5, 110.6, 128.3, 128.4, 128.5, 128.6, 129.6, 129.7, 133.3, 133.5, 135.1, 150.0, 150.1, 163.5, 163.6, 166.3, 166.4.

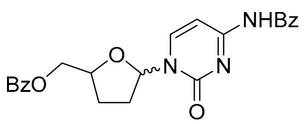

13b

{5-[4-(benzoylamino)-2-oxopyrimidin-1(2*H*)-yl]oxolan-2-yl}methyl benzoate (13b). DCM/MeOH = 99:1; 70% yield; see Schemes 4 and 7. NMR data were fully in agreement with those reported elsewhere [80]. ^{1}H NMR (α:β = 1:2; 400 MHz): δ 1.62-1.80 (*m*, 0.33H), 1.80-2.00 (*m*, 0.66H), 2.01-2.38 (*m*, 2H), 2.57-2.70 (*m*, 0.33H), 2.71-2.82 (*m*, 0.66H), 4.41 (*dd*, J = 5.4, 11.9, 0.66H), 4.48 (*dd*, J = 3.8, 11.9, 0.66H), 4.51-4.59 (*m*, 0.33H), 4.62-4.73 (*m*, 0.66H), 4.77-4.86 (*m*, 0.66H), 6.10 (*dd*, J = 1.9, 6.4, 0.33H), 6.17 (*dd*, J = 3.3, 6.1, 0.66H), 7.42-7.57 (*m*, 3H), 7.58-7.69 (*m*, 1H), 7.92-8.03 (*m*, 2H), 8.05 (*d*, J = 7.1, 1H). ^{13}C NMR (α:β = 1:2; 100 MHz): ppm 24.9, 25.9, 32.9, 33.4, 64.6, 66.0, 79.7, 80.6, 88.7, 89.8, 128.6, 128.8, 129.1, 129.6, 129.7, 133.4, 133.6, 133.7, 165.8,9, 166.2, 172.0.

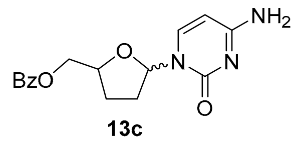

13c

{5-[4-amino-2-oxopyrimidin-1(2*H*)-yl]oxolan-2-yl}methyl benzoate (**13c**). DCM/MeOH = 98:2; >95% yield; see Scheme 7. NMR data were fully in agreement with those reported elsewhere [81]. ^{1}H NMR (α:β = 1:1; 400 MHz): δ 1.79-1.91 (*m*, 0.5H), 1.92-2.04 (*m*, 0.5H), 2.08-2.22 (*m*, 2H), 2.46-2.57 (*m*, 0.5H), 2.58-2.68 (*m*, 0.0.5H), 4.36 (*dd*, J = 5.6, 11.9, 0.5H), 4.39-4.50 (*m*, 1H), 4.60 (*d*, J = 3.8, 1H), 4.71-4.78 (*m*, 0.5H), 5.99 (*dd*, J = 2.9, 6.5, 0.5H), 6.06 (*dd*, J = 3.1, 5.6, 0.5H), 6.10 (*d*, J = 7.5, 0.5H), 6.35 (*d*, J = 7.5, 0.5H), 7.43-7.53 (*m*, 2H), 7.53-7.65 (*m*, 1H), 7.86 (*d*, J = 7.5, 1H), 7.98-8.01 (*m*, 2H). ^{13}C NMR (α:β = 1:1; 125 MHz): ppm 25.4, 26.1, 32.8, 33.1, 64.9, 79.8, 83.3, 87.7, 94.7, 128.6, 128.7, 129.5, 129.7, 133.4, 133.7, 141.3, 153.1, 162.7, 162.9, 166.1.

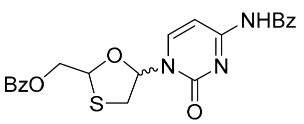

14a

{5-[4-(benzoylamino)-2-oxopyrimidin-1(2H)-yl]-1,3-oxathiolan-2-yl}methyl benzoate (14a). DCM/MeOH = 98:2; 81% yield; see Scheme 4. NMR data were fully in agreement with those reported elsewhere [45,78]. ^{1}H NMR (α:β = 1:1; 400 MHz): δ 3.32-3.45 (*m*, 1H), 3.65-3.82 (*m*, 1H), 4.47 (*dd*, J = 4.2, 12.3, 0.5H), 4.54 (*dd*, J = 6.3, 12.3, 0.5H), 4.81 (*dd*, J = 2.0, 13.0, 0.5H), 4.97 (*dd*, J = 4.4, 13.0, 0.5H), 5.57 (*bs*, 0.5H), 5.97 (*dd*, J = 4.4, 6.3, 0.5H), 6.38 (*d*, J = 4.2, 0.5H), 6.58 (*d*, J = 4.8, 0.5H), 7.40-7.69 (*m*, 7H), 7.91 (*d*, J = 7.6, 2H), 8.04-8.18 (*m*, 3H), 8.26 (*d*, J = 7.6, 1H). ^{13}C NMR (α:β = 1:1; 100 MHz): ppm 29.7, 37.2, 39.3, 63.6, 66.2, 85.5, 86.9, 87.9, 88.2, 90.4, 96.5, 128.6, 128.8, 129.1, 129.4, 129.8, 129.9, 133.5, 133.8, 134.3, 134.7, 147.6, 148.7, 160.9, 163.0, 170.9.

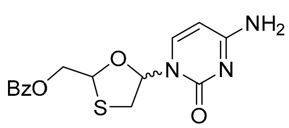

14b

{5-[4-amino-2-oxopyrimidin-1(2*H*)-yl]-1,3-oxathiolan-2-yl}methyl benzoate (**14b**) DCM/MeOH = 95:5; 85% yield; see Scheme 7. NMR data were fully in agreement with those reported elsewhere [82]. ^{1}H NMR (α:β = 1:2;400 MHz): δ 3.12 (*dd*, J = 4.1, 12.2, 0.66H), 3.25 (*dd*, J = 2.0, 12.3, 0.33H), 3.57 (*dd*, J = 5.4, 12.2, 0.66H), 3.61 (*dd*, J = 5.3, 12.3, 0.33H), 4.42 (*dd*, J = 4.3, 11.9, 0.33H), 4.47 (*dd*, J = 6.3, 11.9, 0.33H), 4.70 (*dd*, J = 3.4, 12.3, 0.66H), 4.77 (*dd*, J = 5.1, 12.3 Hz, 0.66H), 5.48 (*dd*, J = 3.4, 5.1 Hz, 0.66H), 5.67 (*d*, J = 7.5, 0.66H), 5.78 (*d*, J = 7.5, 0.33H), 5.79-5.83 (*m*, 0.33H), 6.38 (*dd*, J = 4.1, 5.4, 0.66H), 6.54 (*dd*, J = 2.1, 5.1, 0.33H), 7.49 (*t*, J = 7.6, 1.98H), 7.62 (*t*, J = 7.4, 0.99H), 7.77 (*d*, J = 7.5, 0.99H), 8.08 (*d*, J = 7.1, 1.98H). ^{13}C NMR (α:β = 1:2; 100 MHz): δ 37.6, 38.2, 64.6, 66.6, 83.3, 84.1, 87.5, 88.9, 94.6, 94.9, 128.6, 129.3, 129.7, 133.5, 133.6, 140.5, 140.6, 155.6, 155.7, 165.9, 166.0, 173.3.

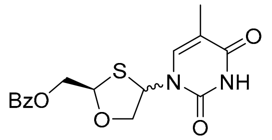

15a

{(2*R*)-4-[5-methyl-2,4-dioxo-3,4-dihydropyrimidin-1(2*H*)-yl]-1,3-oxathiolan-2-yl}methyl benzoate (**15a**). DCM/MeOH = 95:5; 72-75% yield; see Schemes 4 and 7. NMR data were fully in agreement with those reported elsewhere [61]. ^{1}H NMR (α:β = 1:2; 400 MHz): δ 1.76 (*s*, 3H), 1.94 (*s*, 0.33H), 4.01 (*dd*, J = 4.9, 11.0, 0.66H), 4.26 (*dd*, J = 3.3, 12.3, 0.33H), 4.33 (*bs,* 0.33H), 4.47 (*d*, J = 11.0,

0.66H), 4.61 (*dd*, J = 8.3, 12.3, 0.33H), 4.71 (*dd*, J = 5.5, 12.4, 0.66H), 4.83 (*dd*, J = 3.1, 12.4, 0.66H), 5.47 (*dd*, J = 3.1, 12.4, 0.66H), 5.94 (*dd*, J = 3.1, 12.2, 0.33H), 6.50-6.55 (*m*, 1H), 7.41-7.54 (*m*, 3H), 7.61 (*t*, J = 7.4, 1H), 8.06 (*d*, J = 7.4, 2H). ^{13}C NMR (α:β = 1:2; 100 MHz): 12.5, 12.6, 62.5, 62.6, 64.1, 64.4, 75.1, 77.5, 84.3, 85.7, 112.3, 112.4, 128.5, 128.6, 129.8, 133.6, 136.0, 150.3, 150.4, 162.9, 166.0.

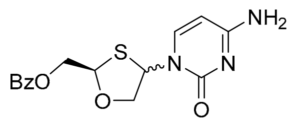

15b

{(2*R*)-4-[4-amino-2-oxopyrimidin-1(2*H*)-yl]-1,3-oxathiolan-2-yl}methyl benzoate (15b). DCM/MeOH = 93:7; 67% yield, α:β = 1:2; see Scheme 8. NMR data were fully in agreement with those reported elsewhere [59]. ^{1}H NMR (α:β = 1:2; 500 MHz): 4.00 (*dd*, J = 4.1, 10.3, 0.66H), 4.22-4.33 (*m*, 0.99H), 4.43 (*d*, J = 10.3, 0.66H), 4.56 (*dd*, J = 8.3, 12.2, 0.33H), 4.70 (*dd*, J = 5.1, 12.3, 0.66H), 4.77 (*dd*, J = 3.2, 12.3, 0.66H), 5.45 (*bs*, 0.66H), 5.58 (*d*, J = 7.5, 0.66H), 5.82 (*d*, J = 7.5, 0.33H), 5.83-5.91 (*m*, 0.33H), 6.62 (b*s*, 1H), 7.47 (*t*, J = 7.4, 2H), 7.62 (*t*, J = 7.4, 1H), 7.66 (*d*, J = 7.5, 0.33H), 7.87 (*d*, J = 7.5, 0.66), 8.08 (*d*, J = 7.1, 2H). ^{13}C NMR (α:β = 1:2; 100 MHz): 63.8, 63.9, 64.4, 64.7, 75.7, 78.3, 84.2, 85.6, 95.9, 96.1, 12836, 128.7, 129.4, 129.5, 129.8, 129.9, 133.5, 133.7, 142.1, 142.3, 155.6, 155.7, 165.0, 165.1, 166.1, 166.2, 173.0.

Synthesis of apricitabine

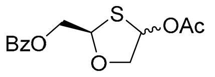

18

[(2*R*)-4-(acetyloxy)-1,3-oxathiolan-2-yl]methyl benzoate (18). A mixture of **17** (0.2 g, 0.87 mmol) and tetrabutylammonium acetate (0.34 g, 1.14 mmol) in acetic anhydride (5 ml) was heated at 120 °C for 14h. The excess of Ac_2O was removed under reduced pressure. Chromatography of the crude residue over silica gel (hexane:EtOAc = 9:1) provided **17** as a diastereoisomeric mixture (0.17 g, 1.5:1 = α:β, 70%). NMR data were fully in agreement with those reported elsewhere [61]. ^{1}H NMR (α:β = 1:1.5; 400 MHz): δ 2.04 (*s*, 1.2H), 2.09 (*s*, 1.8H), 3.92 (*dd*, J = 3.3, 11.8, 0.4H), 4.25 (*dd*, J = 4.2, 11.8, 0.6H), 4.18 (*dd*, J = 3.7, 11.8, 0.4H), 4.33 (*dd*, J = 1.2, 11.8, 0.6H), 4.48-4.62 (*m*, 2H), 5.48 (*dd*, J = 4.5, 0.4H), 5.73 (*dd*, J = 3.6, 8.0, 0.6H), 6.22 (*d*, J = 3.8, 0.4H), 6.27 (*dd*, J = 1.2, 4.2, 0.6H), 7.36-7.48 (*m*, 2H), 7.51-7.56 (*m*, 1H), 8.06-8.09 (*m*, 2H). ^{13}C NMR (α:β = 1:1.5; 100 MHz): ppm 21.0, 64.7, 66.1, 74.4, 76.9, 81.1, 81.2, 83.8, 84.5, 128.3, 129.8, 133.3, 166.1, 170.4, 171.1.

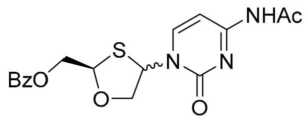

15c

{(2*R*)-4-[4-(acetylamino)-2-oxopyrimidin-1(2*H*)-yl]-1,3-oxathiolan-2-yl}methyl benzoate (15c). Nucleoside **15c** was obtained according to the *N*-glycosidation reaction, general procedure (Step 1, rt, 24h; step 2, rt, 24h) as α:β = 1:4 anomeric mixture (70% yield). Chromatography of the crude residue over silica gel (hexane:EtOAc = 3:7) provided the pure α- and β-anomers as white solids. NMR data were fully in agreement with those reported elsewhere [59,61,62]. Data for β-anomer. ^{1}H NMR (400 MHz): δ 2.23 (*s*, 3H), 4.06 (*dd*, J = 4.1, 10.5, 1H), 4.48 (*d,* J =10.5, 1H), 4.75 (*dd*, J = 4.3, 12.6, 1H), 4.86 (*dd*, J· = 3.0, 12.6, 1H), 5.48 (*bs*, 1H), 6.63 (*d*, J = 4.0, 1H), 7.22 (*d*, J = 7.5, 1H), 7.40-7.75 (*m*, 4H), 8.08 (*d*, J = 8.1 Hz, 2H), 8.29 (*d*, J = 7.5 Hz, 1H, H-6), 8.89 (*bs*, 1H). ^{13}C NMR (100 MHz): 25.9, 64.3, 65.3, 78.7, 86.2, 97.9, 128.5, 128.7, 129.2, 129.4, 129.8, 133.7, 145.6, 155.3, 162.5, 162.7, 171.0. Data for α-anomer. ^{1}H NMR (400 MHz, DMSO-d_6): δ 2.06 (*s*, 3H), 4.31 (*dd*, J = 3.1, 12.5, 1H), 4.72 (*dd*, J = 8.3, 12.5, 1H), 5.92 (*dd*, J =3.2 Hz, J = 8.1 Hz, 1H), 6.35 (*bs*, 1H), 7.20 (*d*, J = 7.5, 1H), 7.21 (*d*, J = 7.5, 1H), 7.40-7.75 (m, 4H), 8.08 (*d*, J = 8.1 Hz, 2H), 8.29 (d, J = 7.5 Hz, 1H, H-6), 8.89 (*bs*, 1H). ^{13}C NMR (100 MHz): 25.9, 65.1, 65.3, 76.1, 84.7, 98.1, 128.5, 128.7, 129.2, 129.4, 129.8, 133.5, 145.3, 155.2, 162.5, 162.6, 171.

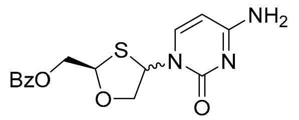

Apricitabine

{(2*R*)-4-[4-amino-2-oxopyrimidin-1(2*H*)-yl]-1,3-oxathiolan-2-yl}methyl benzoate (Apricitabine). MeONa (14.0 mg, 0.26 mmol) was added to a stirred solution of **15c** (0.10 g, 0.26 mmol, β-anomer) in MeOH (13 mL) under argon atmosphere at rt. The reaction mixture was stirred at the same temperature for 6h and then glacial acetic acid was added until pH ~ 5. MeOH was removed under reduced pressure and the crude was purified by chromatography over silica gel ($CHCl_3$/MeOH = 9:1) to give pure apricitabine (57 mg, 96% yield). NMR data were fully in agreement with those reported elsewhere [59,63]. $[\alpha]_D^{25}$ = -76.0 (c 1.1); white solid. ^1H NMR (400 MHz, DMSO-d_6): δ 3.65-3.85 (*m*, 2H), 4.08 (*d,* J =11.3, 1H), 4.20 (*d*, J = 11.2, 1H), 5.10 (*t*, J = 4.9, 1H), 5.48 (*t*, J =7.0, 1H), 5.80 (*d*, J = 7.4, 1H), 6.33 (*d*, J = 3.7, 1H), 7.75 (*d*, J = 7.4 Hz, 1H). ^{13}C NMR (100 MHz, DMSO-d_6): ppm 62.9, 63.0, 77.6, 89.0, 95.4, 142.4, 155.7, 165.9.

Copies of NMR Spectra

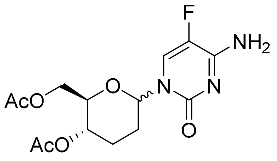

10d

^{1}H NMR (400 MHz, $CDCl_3$)

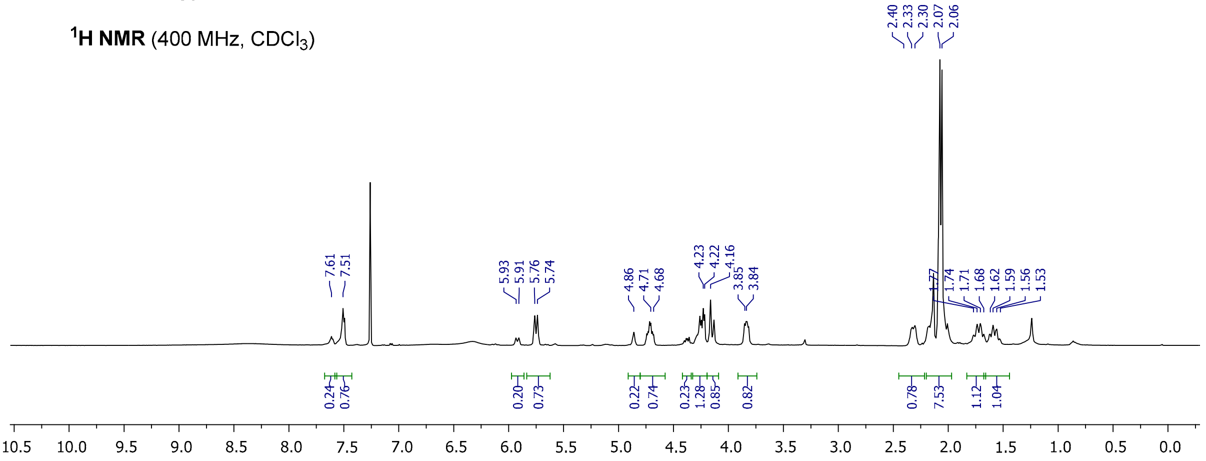

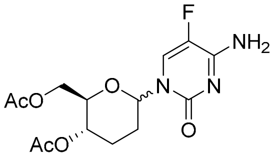

10d

^{13}C NMR (100 MHz, $CDCl_3$)

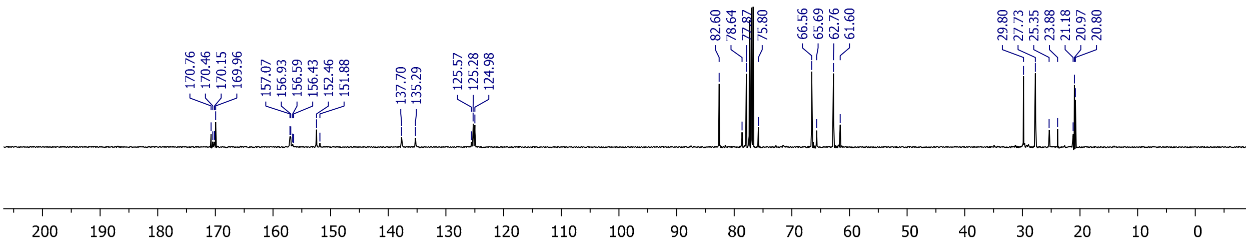

Figure S1. Copies of ^{1}H and ^{13}C NMR spectra for compound **10d**.

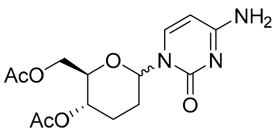

10e

^{1}H NMR (400 MHz, $CDCl_3$)

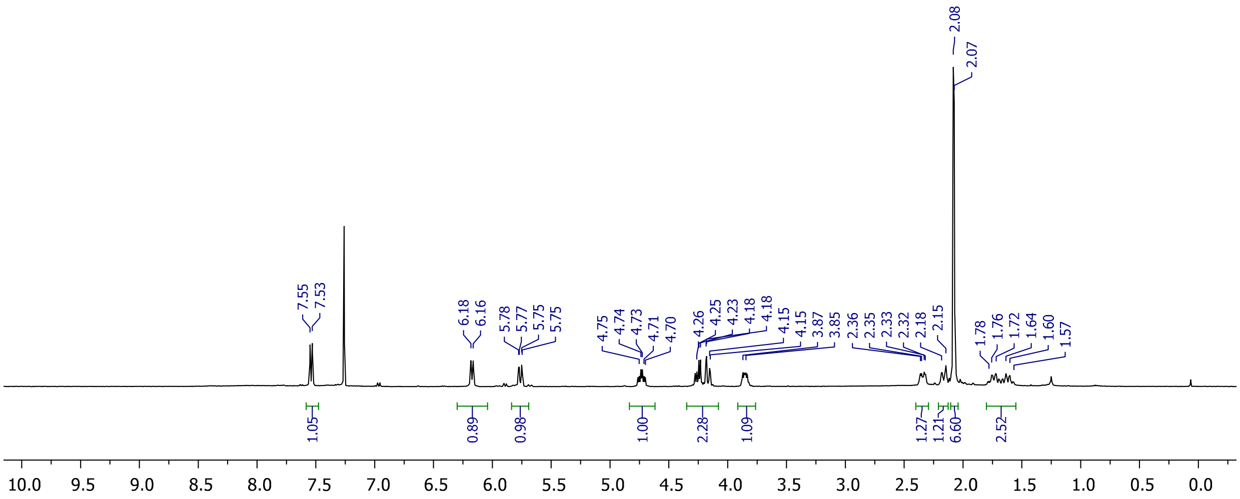

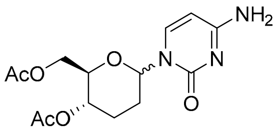

10e

^{13}H NMR (100 MHz, $CDCl_3$)

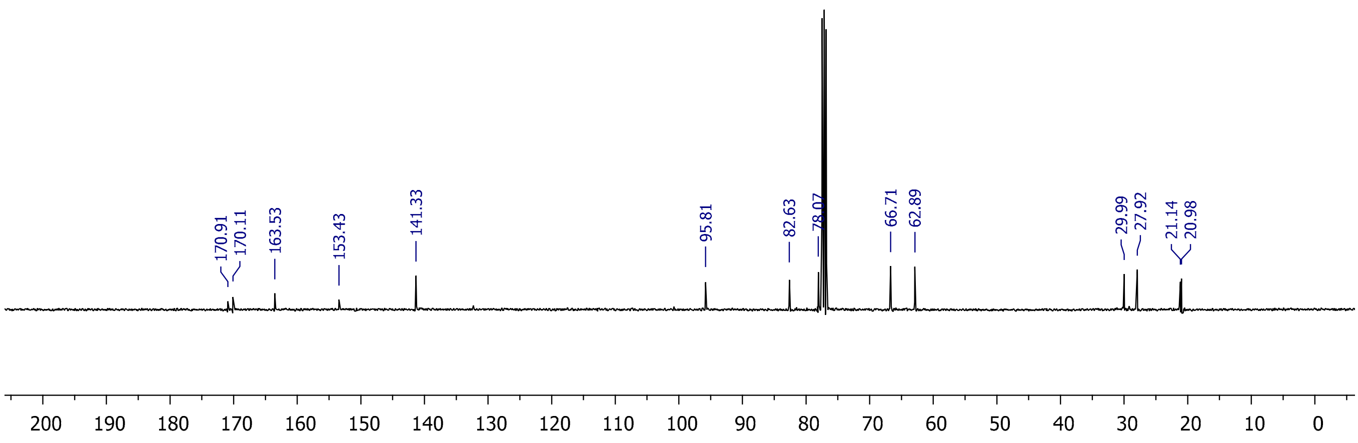

Figure S2. Copies of ^{1}H and ^{13}C NMR spectra for compound **10e**.

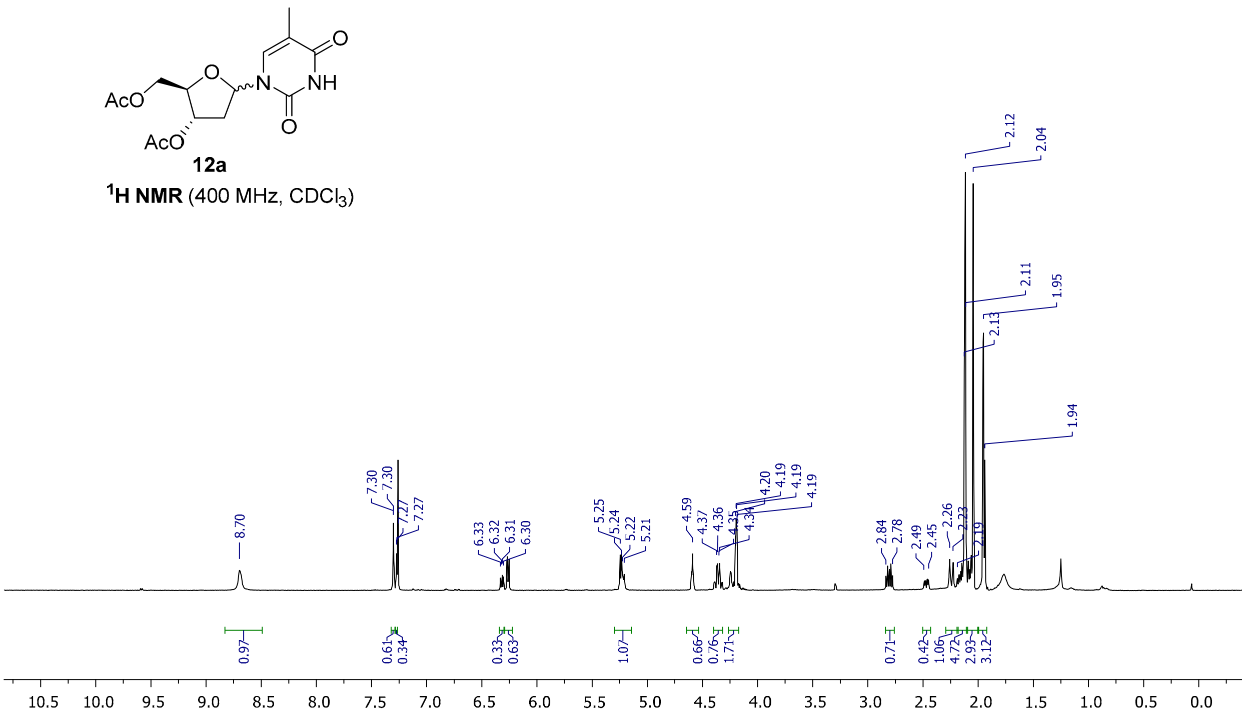

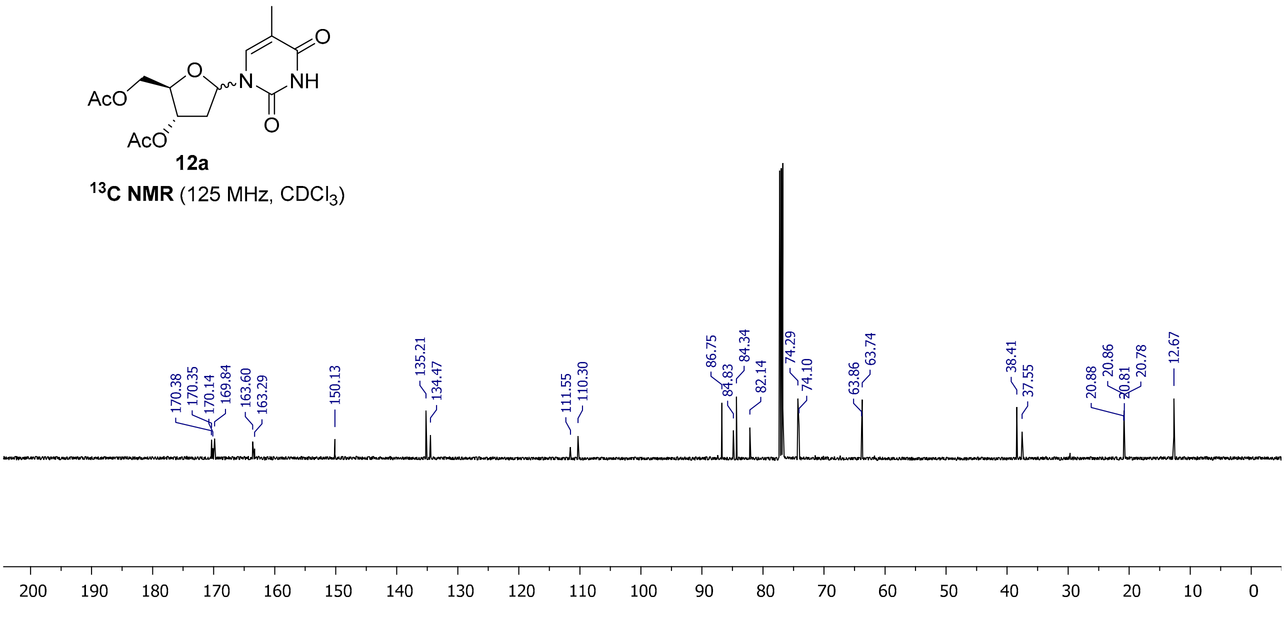

Figure S3. Copies of ^{1}H and ^{13}C NMR spectra for compound **12a**.

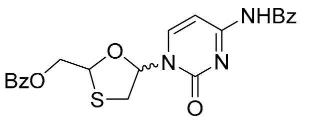

14a

¹H NMR (400 MHz, $CDCl_3$)

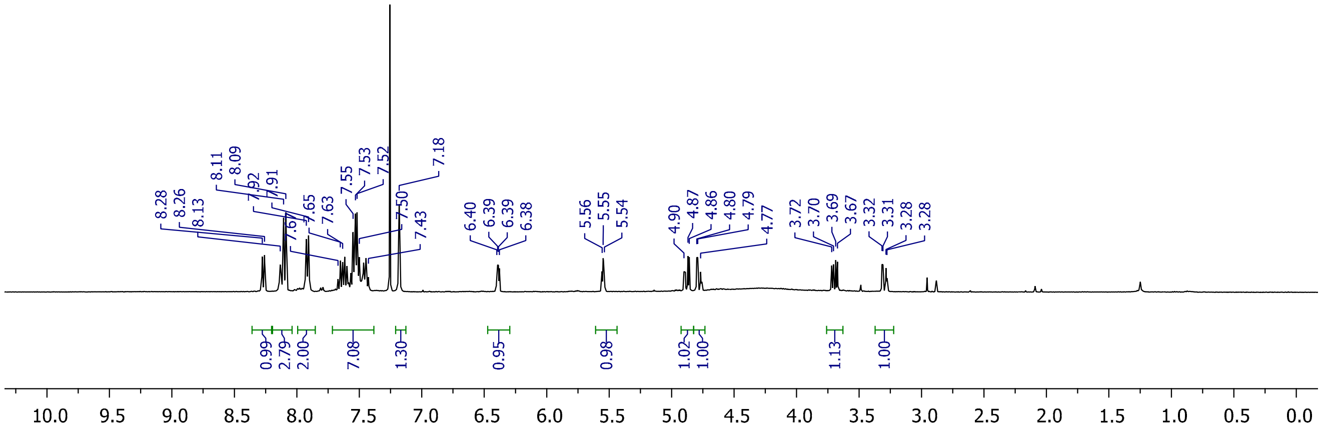

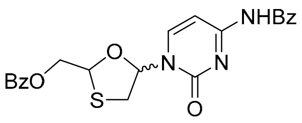

14a

¹³C NMR (100 MHz, $CDCl_3$)

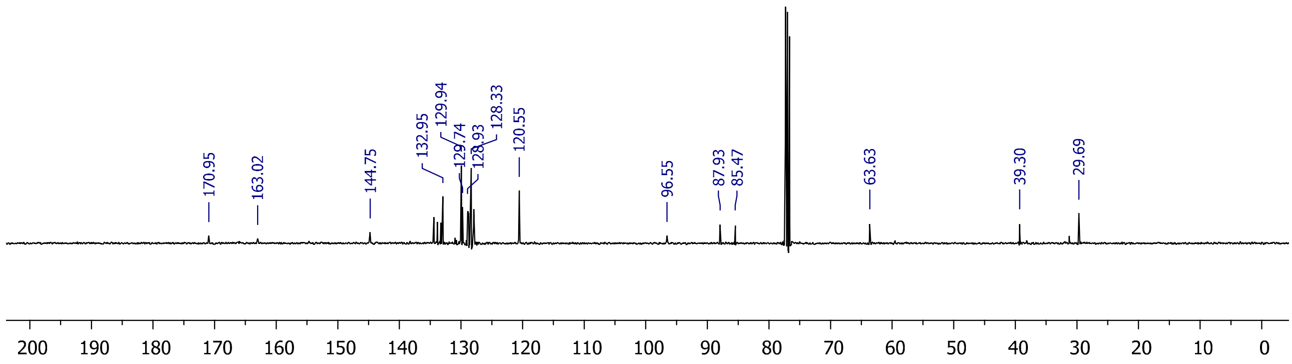

Figure S4. Copies of ^{1}H and ^{13}C NMR spectra for compound **14a**.

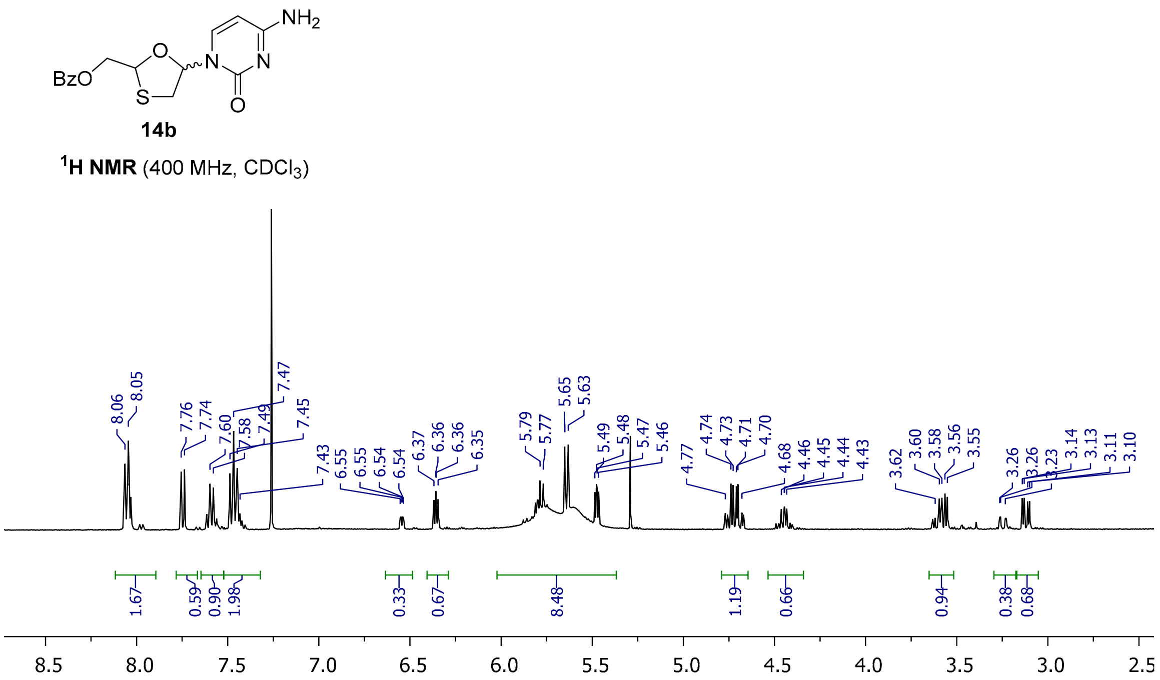

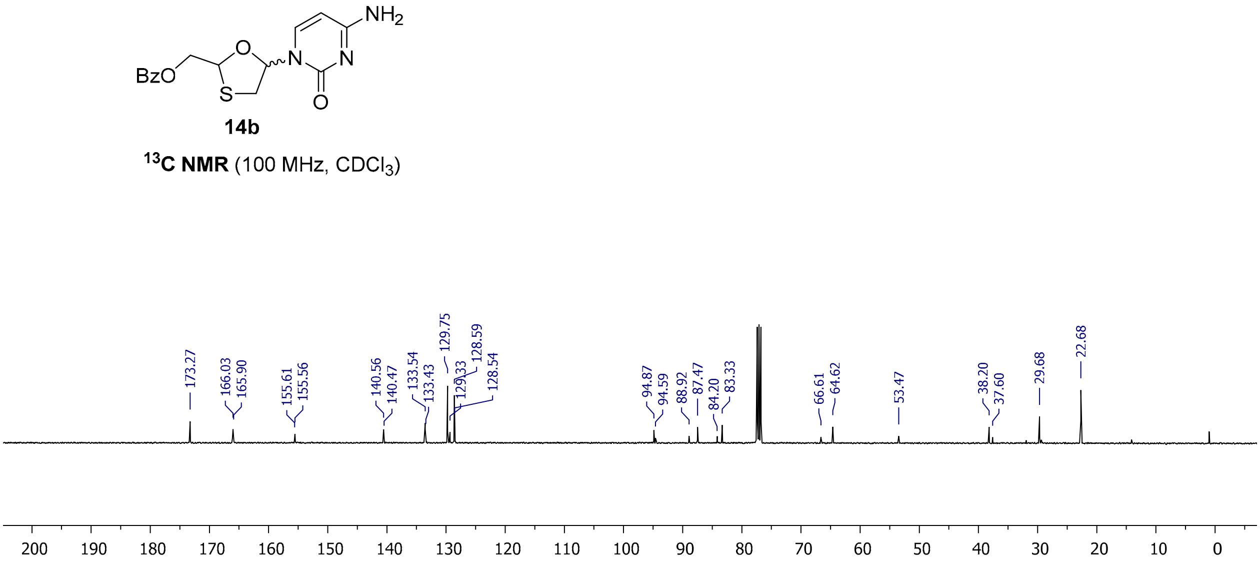

Figure S5. Copies of ¹H and ¹³C NMR spectra for compound **14b**.

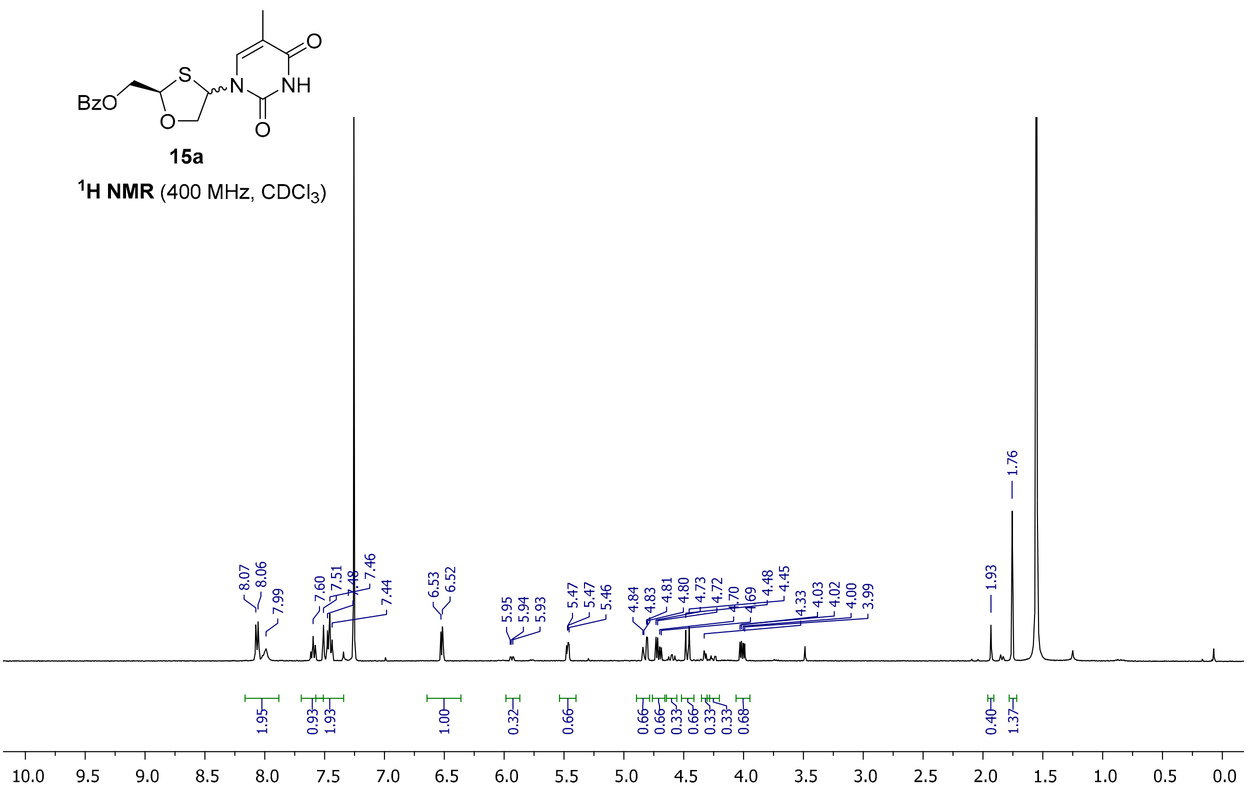

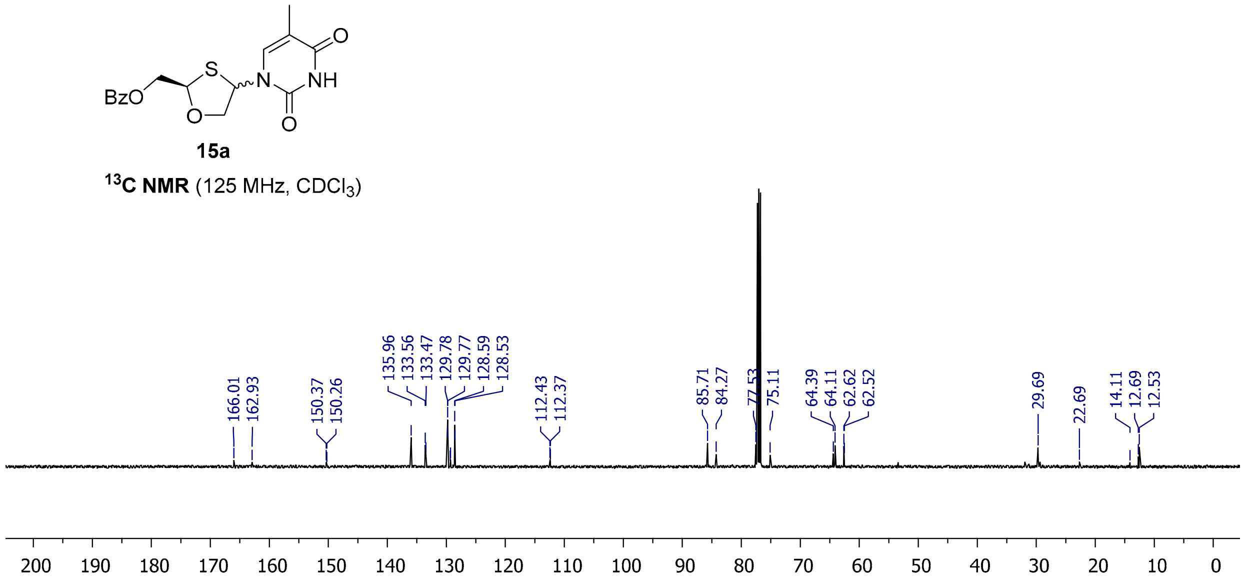

Figure S6. Copies of ^{1}H and ^{13}C NMR spectra for compound **15a**.

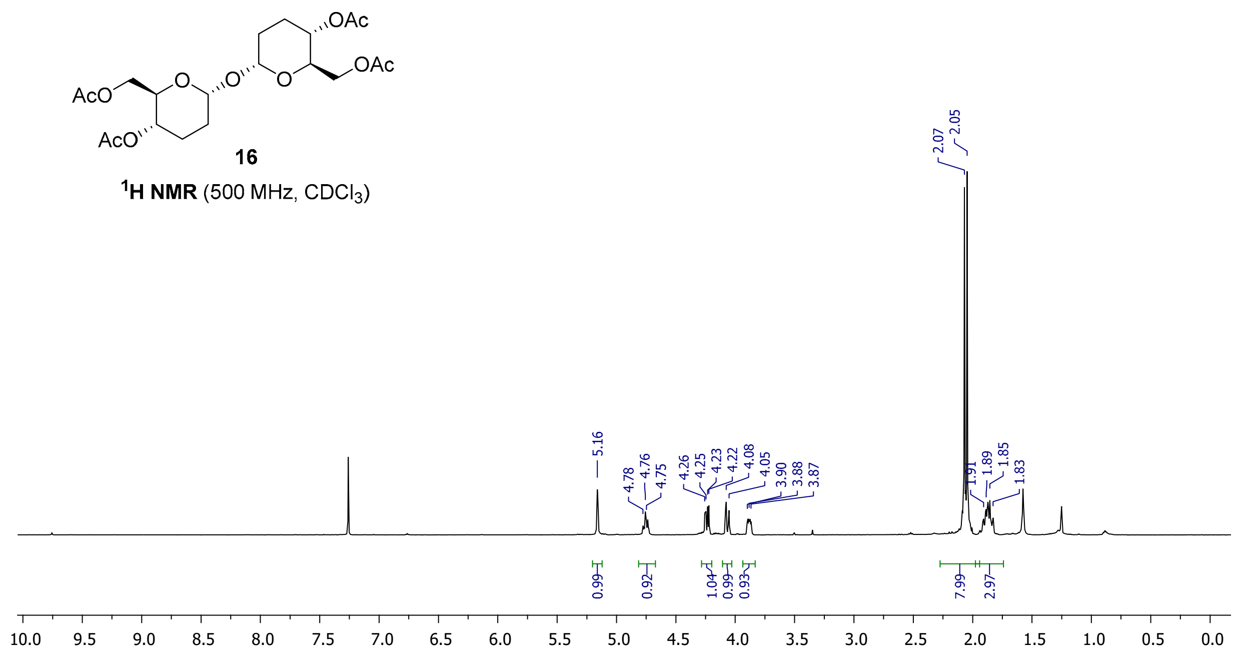

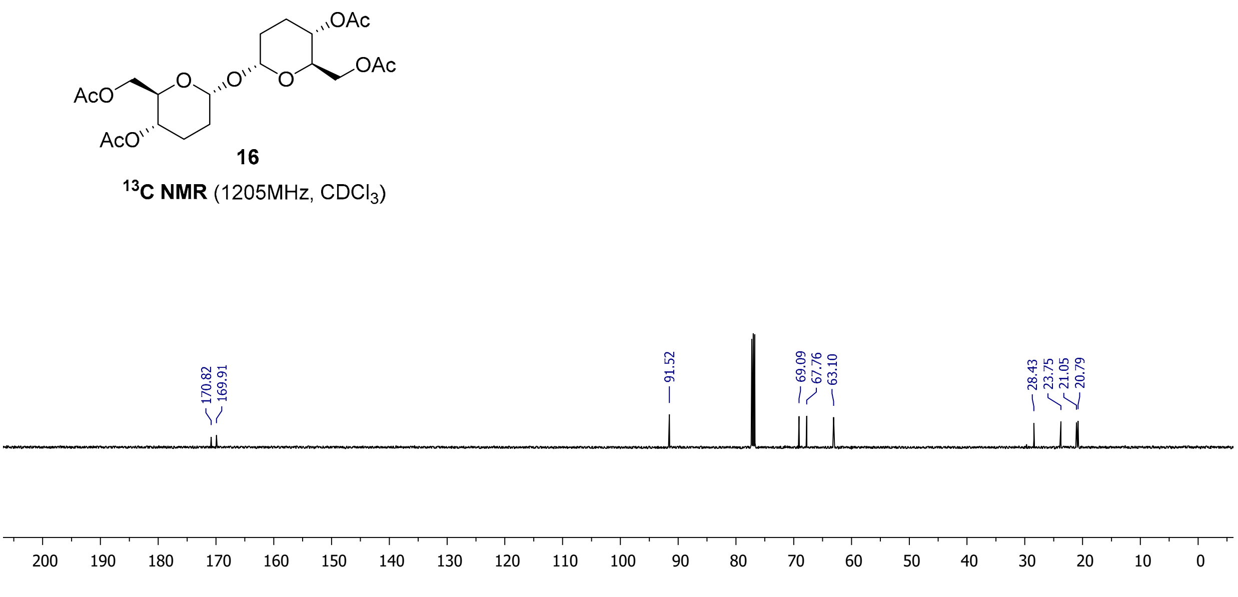

Figure S7. Copies of ^{1}H and ^{13}C NMR spectra for compound **16**.

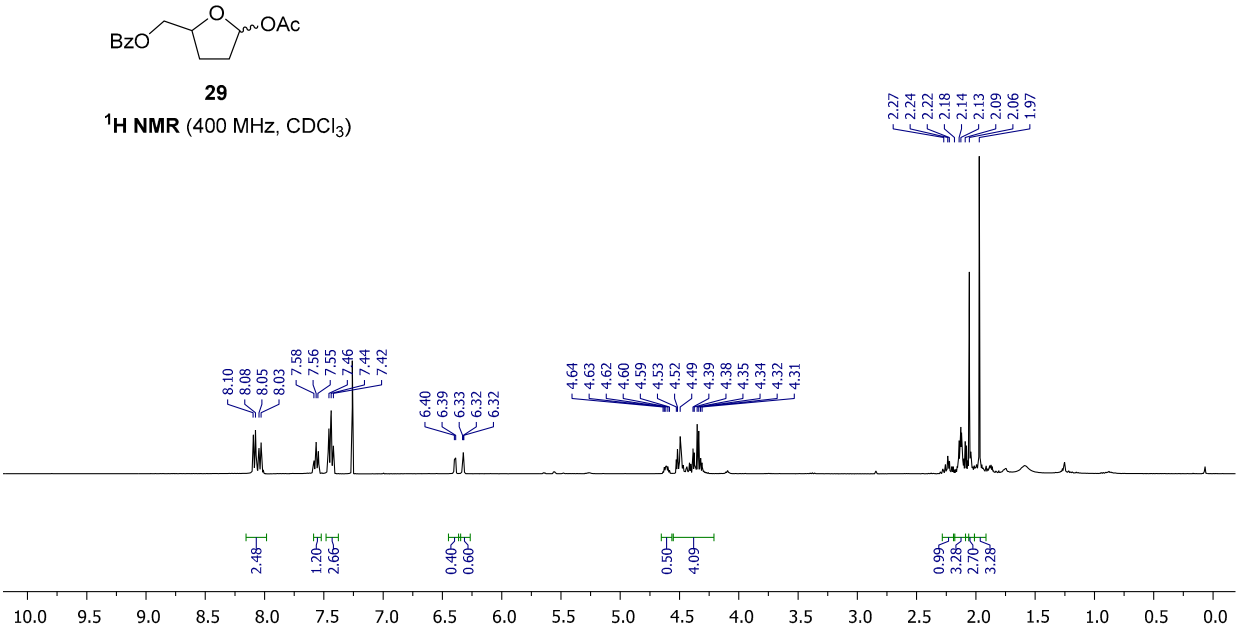

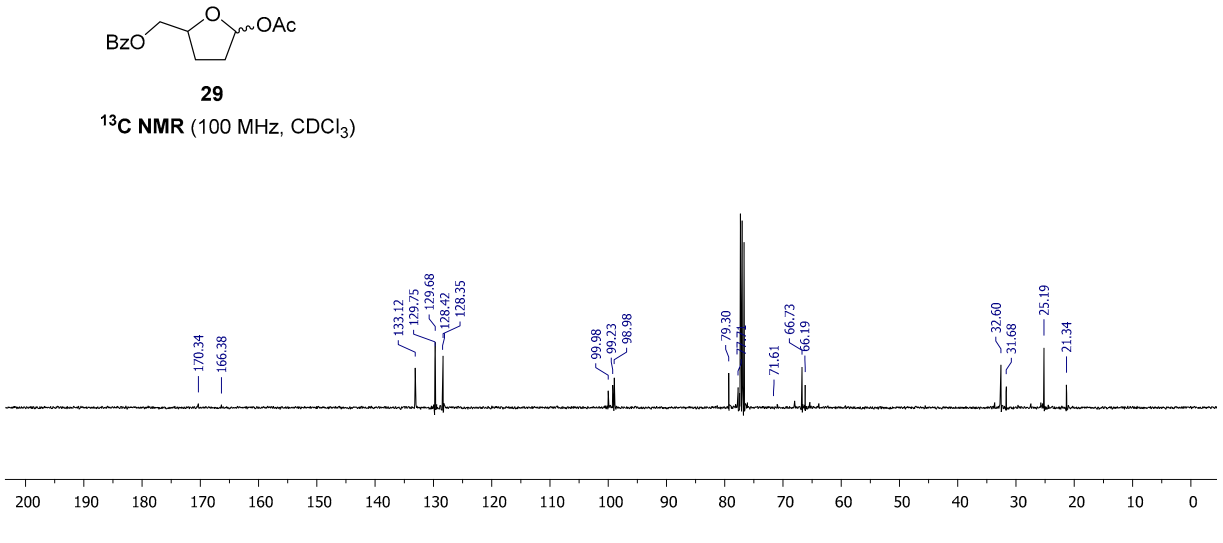

Figure S8. Copies of ^{1}H and ^{13}C NMR spectra for compound **29**.

Bibliography

60. Belleau, B.; Nguyen-ba, N.; Kong, L.C.C.; Mansour, T.; Jin, H.; Brasili, L. Substituted 1,3-Oxathiolanes and Substituted 1,3-Dithiolanes with Antiviral Properties **1996.**
61. Mansour, T.; Haulon, J. Substituted 1,3-Oxathiolanes with Antiviral Properties. Eur. Pat. 1473294 A2 **2004**, 1.
62. Mansour, T.S.; Jin, H.; Wang, W.; Hooker, E.U.; Ashman, C.; Cammack, N.; Salomon, H.; Belmonte, A.R.; Wainberg, M.A. Anti-Human Immunodeficiency Virus and Anti-Hepatitis-B Virus Activities and Toxicities of the Enantiomers of 2′-Deoxy-3′-Oxa-4′-Thiocytidine and Their 5-Fluoro Analogs in Vitro. *J. Med. Chem.* **1995**, *38*, 1–4, doi:10.1021/jm00001a001.
63. Ludek, O.R.; Marquez, V.E. A Greener Enantioselective Synthesis of the Antiviral Agent North-Methanocarbathymidine (N-MCT) from 2-Deoxy-d-Ribose. *Tetrahedron* **2009**, *65*, 8461–8467, doi:10.1016/j.tet.2009.08.035.
64. Jun, S.J.; Moon, M.S.; Lee, S.H.; Cheong, C.S.; Kim, K.S. Selective Monodeacetylation of Methyl 2,3,5-Tri-O-Acetyl-d-Arabinofuranoside Using Biocatalyst. *Tetrahedron Lett.* **2005**, *46*, 5063–5065, doi:10.1016/j.tetlet.2005.05.120.
65. Taverna-Porro, M.; Bouvier, L.A.; Pereira, C.A.; Montserrat, J.M.; Iribarren, A.M. Chemoenzymatic Preparation of Nucleosides from Furanoses. *Tetrahedron Lett.* **2008**, *49*, 2642–2645, doi:10.1016/j.tetlet.2008.02.087.
66. Koreeda, M.; Houston, T.A.; Shull, B.K.; Klemke, E.; Tuinman, R.J. Iodine-Catalyzed Ferrier Reaction 1. A Mild and Highly Versatile Glycosylation of Hydroxyl and Phenolic Groups1. *Synlett* **1995**, *1995*, 90–92, doi:10.1055/s-1995-4873.
67. Sasaki, K.; Wakamatsu, T.; Matsumura, S.; Toshima, K. Synthesis of Hexopyranosyl Acetates and 2,3-Disubstituted Tetrahydropyrans via Chemoselective Hydrogenation of Hex-2-Enopyranosyl Acetates. *Tetrahedron Lett.* **2006**, *47*, 8271–8274, doi:10.1016/j.tetlet.2006.09.091.
68. Regueira, J.; Dantas, C.; de Freitas, J.; da Silva, A.; Freitas Filho, J.; Menezes, P.; Freitas, J. Stereoselective Synthesis of 2,3-Unsaturated Pseudoglycosides Promoted by Ultrasound. *Synthesis (Stuttg).* **2016**, *48*, 1069–1078, doi:10.1055/s-0035-1561344.
69. Banaag, A.R.; Tius, M.A. Traceless Chiral Auxiliaries for the Allene Ether Nazarov Cyclization. *J. Org. Chem.* **2008**, *73*, 8133–8141, doi:10.1021/jo801503c.
70. Jung, S.; Inoue, A.; Nakamura, S.; Kishi, T.; Uwamizu, A.; Sayama, M.; Ikubo, M.; Otani, Y.; Kano, K.; Makide, K.; et al. Conformational Constraint of the Glycerol Moiety of Lysophosphatidylserine Affords Compounds with Receptor Subtype Selectivity. *J. Med. Chem.* **2016**, *59*, 3750–3776, doi:10.1021/acs.jmedchem.5b01925.
71. Sarkar, B.; Pramanik, T.; Jayaraman, N. Cyclic Disaccharide Formation Enforced by a Ring Contraction: 2,3-Dideoxy Pyranoside Glycoside Donor to a Furanoside Macrocycle. *J. Org. Chem.* **2023**, *88*, 670–674, doi:10.1021/acs.joc.2c01936.
72. Lu, Y.-S.; Li, Q.; Zhang, L.-H.; Ye, X.-S. Highly Direct α-Selective Glycosylations of 3,4- O -Carbonate-Protected 2-Deoxy- and 2,6-Dideoxythioglycosides by Preactivation Protocol. *Org. Lett.* **2008**, *10*, 3445–3448, doi:10.1021/ol801190c.
73. Yakura, T.; Horiuchi, Y.; Nishimura, Y.; Yamada, A.; Nambu, H.; Fujiwara, T. Efficient Oxidative Cleavage of Tetrahydrofuran-2-methanols to Γ-Lactones by a 2-Iodobenzamide Catalyst in Combination with Oxone ®. *Adv. Synth. Catal.* **2016**, *358*, 869–873, doi:10.1002/adsc.201500795.
74. Hu, L.; Schaufelberger, F.; Zhang, Y.; Ramström, O. Efficient Asymmetric Synthesis of Lamivudine via Enzymatic Dynamic Kinetic Resolution. *Chem. Commun.* **2013**, *49*, 10376–10378, doi:10.1039/C3CC45551C.
75. Böhringer, M.; Roth, H.; Hunziker, J.; Göbel, M.; Krishnan, R.; Giger, A.; Schweizer, B.; Schreiber, J.; Leumann, C.; Eschenmoser, A. Oligonucleotide Aus 2′,3′-Dideoxy-β-D-glucopyranosyl-Bausteinen ('Homo-DNS'): Herstellung. *Helv. Chim. Acta* **1992**, *75*, 1416–1477, doi:10.1002/hlca.19920750503.
76. Khan, A.R.; Mulligan, K.X.; Redda, K.K.; Ollapally, A.P. Synthesis of 3′-azido-2′,3′- dideoxy-4′-ketohexopyranoid analogues as possible antiviral nucleosides. *Synth. Commun.* **2002**, *32*, 1023–1030, doi:10.1081/SCC-120003150.
77. Chandra, T.; Broderick, W.E.; Broderick, J.B. An Efficient Deprotection of *N* -Trimethylsilylethoxymethyl (SEM) Groups From Dinucleosides and Dinucleotides. *Nucleosides. Nucleotides Nucleic Acids* **2010**, *29*, 132–143, doi:10.1080/15257771003612847.
78. Palomino, E.; Meltsner, B.R.; Kessel, D.; Horwitz, J.P. Synthesis and in Vitro Evaluation of Some Modified 4-Thiopyrimidine Nucleosides for Prevention or Reversal of AIDS - Associated Neurological Disorders. *J. Med. Chem.* **1990**, *33*, 258–263, doi:10.1021/jm00163a043.
79. Jo, K.; Frode, R.; Kjell, U. 2′,3′ Dideoxyribofuranoxide Derivatives U.S. Patent WO1988007532A1, 1988.
80. Kaulinya, L.T.; Liepin'sh; Lidak, M.Y.; Zhuk, R.A. Analogs of Pyrimidine Nucleosides. Racemic 2,3-Dideoxynucleosides and Their Derivatives. *Chem. Heterocycl. Compd.* **1982**, *18*, 85–93, doi:10.1007/BF00513297.
81. Coates, J.A.V.; Mutton, I.M.; Penn, C.R.; Storer, R.; Williamson, C. 1,3-Oxathiolane Nucleoside Analogues 1991.
